# Computational Models for Bilingual Aphasia: A Systematic Review of Language Deficit Research with a Focus on Code-Switching and Translation

**DOI:** 10.3390/bs16091486

**Published:** 2026-08-25

**Authors:** Si Chen, Ruilan Cao, Sijia Cheng

**Affiliations:** 1 School of Foreign Languages, Anhui University of Technology, Ma’anshan 243002, China; cheese_css@ahut.edu.cn; 2 School of International Education, Anhui University of Technology, Ma’anshan 243002, China; chengsijia@ahut.edu.cn

**Keywords:** computational models, bilingual aphasia, systematic review, computational lesion, language control, code-switching, translation abilities, mechanism simulation

## Abstract

This systematic review synthesises computational models of language deficits in bilingual aphasia, focusing on code-switching and translation. Per the Preferred Reporting Items for Systematic Reviews and Meta-Analyses (PRISMA) guidelines, this study identified 42 publications (2010–2025). Of the three deficit types, lexical retrieval and naming deficit simulations are most mature, replicating naming errors and predicting cross-language generalisation. Code-switching and translation deficit modelling is extremely limited: only a single computational lesioning study has addressed both through mechanistic simulation. Machine learning methods show preliminary promise for predicting treatment outcomes but remain largely data-driven. Three key challenges emerge: (1) methodological fragmentation—heterogeneous model types, lesion implementations, and evaluation metrics, with no standardised quantitative validation; (2) an underdeveloped theoretical foundation, as most studies are confined to behavioural fitting rather than testing hypotheses on impaired language control via computational lesions; (3) limited clinical translation, with few predictive studies integrating longitudinal patient data, with approximately 70% of studies concentrated in North America/Western Europe and biased toward English–Spanish bilinguals, limiting theoretical generalisability. Future work should shift from behavioural fitting to explainable mechanistic simulation, develop predictive frameworks for personalised rehabilitation, and establish standardised protocols bridging computational modelling with clinical application.

## 1. Introduction

### 1.1. Research Background

The accelerating global movement of populations and the deepening of cultural integration have rendered the acquisition and use of two or more languages a normative feature of contemporary society (Bak et al., 2014). According to available estimates, over half of the world’s population possesses bilingual or multilingual capabilities in daily life (Bak et al., 2014). This linguistic reality means that, when stroke or other acquired brain injuries occur, a substantial proportion of aphasic patients encountered by clinicians are bilingual speakers (Y.-Y. Lee, 2022; Russell-Meill et al., 2025). Concurrently, global demographic ageing and persistently high stroke incidence rates further compound the clinical burden of bilingual aphasia, elevating it to an increasingly prominent public health concern (Feigin et al., 2014). Bilingual aphasia refers to aphasia occurring in individuals who, based on clinical criteria such as the ICD-11 or DSM-5, are proficient in two or more languages and attain a level of functional communication, that is, the ability to use both languages in everyday interactions (Ansaldo & Saidi, 2014; Russell-Meill et al., 2025). In comparison with monolingual aphasia, the language impairments exhibited by bilingual aphasic patients typically manifest more intricate patterns, a complexity attributable not only to the coexistence of two linguistic systems but also, more critically, to disruptions in the cross-language control mechanisms responsible for language selection, inhibition, and switching under pathological conditions (Abutalebi & Green, 2007; Hameau et al., 2023). Patients may confront a constellation of bilingual-specific, higher-order deficits, including difficulties in code-switching, asymmetries in translation performance, or failures of the language selection apparatus. Although these phenomena constitute vital evidence for clinical diagnosis, the underlying computational principles governing the bilingual brain’s control mechanisms remain, to date, insufficiently elucidated (Nadeau, 2019; Russell-Meill et al., 2025).

### 1.2. Research Status and Problem Formulation

In recent years, a body of representative literature has accumulated at the intersection of artificial intelligence (AI), computational techniques, and aphasia research. These investigations have predominantly focused on applications with direct instrumental utility, including auxiliary diagnosis, automated assessment, pathological speech recognition, rehabilitation management, and treatment outcome prediction, thereby playing a significant role in advancing technological integration into the study of clinical language disorders (Adikari et al., 2024; Russell-Meill et al., 2025). A comprehensive review of these studies, however, reveals that the prevailing discourse has largely conceptualised computational models as engineering tools for classification, prediction, or decision support. A critical yet underexplored question remains as to whether computational models can transcend this instrumental role and serve as an experimental platform for cognitive mechanism simulation, that is, as a means to test mechanistic hypotheses concerning bilingual aphasia within formalised systems (Marte et al., 2025; Russell-Meill et al., 2025).

This issue assumes particular salience in the context of bilingual aphasia research. In contrast to monolingual aphasia, which primarily involves general difficulties in lexical retrieval or naming, bilingual aphasia frequently entails higher-order control deficits, including imbalances in cross-language activation, aberrant language selection, pathological code-switching, translation impairments, and asymmetric recovery patterns (Hameau et al., 2023; Mooijman et al., 2026). Such phenomena suggest that the underlying pathology is not merely attributable to localised loss of linguistic knowledge but more critically involves impairments in cognitive processes such as cross-language competitive inhibition, task switching, language monitoring, and the allocation of control resources (Coemans et al., 2023; Pisano et al., 2022; Sajid et al., 2020). Traditional clinical investigations, which rely primarily on case observations, behavioural assessments, and treatment follow-ups, can provide richly descriptive phenomenological data but are often constrained in their capacity to systematically manipulate latent mechanistic variables and test causal relationships under controlled conditions (Kiran et al., 2013b; Miller Amberber, 2012). Within this context, the importance of computational models becomes increasingly evident. Researchers can simulate functional anomalies in the language processing system following brain injury through controlled manipulations of model parameters, internal representations, modular connectivity, or input conditions. This, in turn, permits the examination of whether a given theoretical mechanism is sufficient to account for specific bilingual pathological manifestations (Sajid et al., 2021; Walker & Hickok, 2016).

To broadly encapsulate such operations, in which functional attenuation, structural perturbation, or processing constraints are deliberately introduced within a model to simulate impaired cognitive states, this review adopts the operational term “computational lesion”, also referred to as an in silico lesion in the broader computational neuroscience literature. It must be clarified that this term is not intended to presuppose a singular technical approach; rather, it is employed as an analytical construct that subsumes the diverse implementation methods used across different modelling paradigms to represent lesioned states (Nadeau, 2019; Sajid et al., 2020). In contrast to the prevailing narrative that foregrounds the application of AI to aphasia as a diagnostic or assistive technology, this review places greater emphasis on the correspondence between a model’s internal processes and the theoretical constructs of cognition. The central concern thus shifts from merely ascertaining whether a model is effective to understanding how the model structurally and procedurally generates specific deficits, and whether this generative process constitutes valid support for mechanistic hypotheses (Grasemann et al., 2021; Walker & Hickok, 2016). The concept of simulating functional deficits through parametric manipulation of a cognitive model has a substantial precedent in monolingual computational neuropsychology. For over three decades, connectionist models of single-word reading and naming have been systematically lesioned by perturbing connection weights, decay rates, or processing pathways to map specific parameter changes onto aphasic error patterns, thereby providing mechanistic accounts of acquired dyslexia and naming impairments (Dell et al., 1997; Foygel & Dell, 2000; Plaut & Shallice, 1993; Welbourne & Lambon Ralph, 2005). The present review extends this lesion-mapping logic to the bilingual case, where the additional dimension of cross-language control introduces qualitatively new modelling challenges beyond those encountered in monolingual architectures.

Although this review adopts an inclusive operational definition of “computational model”, the technical pathways subsumed under this term differ in their epistemological function, and it is useful to acknowledge this from the outset. The literature synthesised herein can be situated within three broad analytical categories, distinguished not primarily by technical architecture but by the epistemic role they perform with respect to bilingual aphasia. The first category comprises mechanism-simulation models. Examples include the BiLex family (Grasemann et al., 2011, 2021; Peñaloza et al., 2019, 2020), the semantic–lexical–auditory–motor (SLAM) model (Walker & Hickok, 2016), and active inference frameworks by Sajid et al. (2020, 2021). These models are designed to test causal hypotheses about impaired bilingual cognitive control through systematic computational lesioning, and they maintain a direct, concept-level mapping to cognitive theory. The second category comprises data-driven classification and prediction models, including traditional machine learning (ML) classifiers, speech recognition systems, and electroencephalography (EEG) decoders. These models are optimised for diagnostic or prognostic accuracy (Adikari et al., 2024; Barbera et al., 2021; Rajendran & Chandrasekar, 2024). They may achieve higher technical performance but typically lack an explicit representational framework for bilingual control and impairment mechanisms. The third comprises general-purpose large language models (LLMs), whose application to bilingual aphasia remains exploratory (Bao et al., 2025) and whose capacity for mechanistic explanation has yet to be demonstrated. These categories are not a strict ontological partition, as individual studies may straddle more than one, but they serve as a navigational aid for interpreting the comparisons that follow.

In the bilingual domain, computational models of lexical processing have a well-established lineage that is directly relevant to understanding the architectures reviewed herein. The bilingual interactive-activation framework (Dijkstra & van Heuven, 2002) introduced the foundational principle of language-nonselective lexical access, wherein orthographic, phonological, and semantic representations from both languages are activated in parallel during word recognition, and language membership is represented by language nodes within an integrated lexicon. The Multilink model (Dijkstra et al., 2019) subsequently extended this tradition to encompass word production and translation, integrating assumptions from both the bilingual interactive activation plus (BIA+) framework and the Revised Hierarchical Model of bilingual memory. The computational architectures examined in the present review, most notably the BiLex model (Grasemann et al., 2010, 2011, 2021; Peñaloza et al., 2019, 2020), which descends directly from this interactive-activation tradition, build upon these foundational frameworks. However, whereas BIA+ and Multilink were developed to characterise lexical processing in the intact bilingual mind, their adaptation to modelling impaired bilingual control, which involves the systematic application of computational lesions to simulate code-switching deficits, translation asymmetries, and pathological language selection, is a comparatively recent and still nascent undertaking. At present, a systematic synthesis specifically addressing how computational models simulate the mechanisms underlying bilingual-specific language deficits through computational lesioning remains notably lacking. Existing contributions tend either to emphasise performance evaluation at the clinical tool level or to remain confined to high-level methodological discourse, seldom integrating the following interrelated dimensions: the technical genealogy of disparate model types, the concrete implementation pathways for computational lesions, the nature of deficits modelled and their bilingual specificity, the strength of evidence marshalled in support of mechanistic interpretations, and the reciprocal relationship between these studies and cognitive neuroscience theories (Russell-Meill et al., 2025; Senadheera et al., 2024). Consequently, the field is in pressing need of a systematic review centred on mechanistic simulation to ascertain the extent to which computational models have progressed beyond mere behavioural fitting to become effective research instruments for deciphering the complex mechanisms of bilingual aphasia.

Grounded in this identified lacuna, the present systematic review is conducted with the objective of systematically consolidating and evaluating existing research that employs computational lesions to simulate specific language deficits in bilingual aphasia from a computational modelling perspective. The central aim of this review is to provide methodological guidance and recommendations for future research directions through a critical synthesis of the available evidence. Furthermore, this review seeks to assess the progress and limitations of these simulations in terms of mechanistic explanation, theoretical dialogue, and clinical implications (Nadeau, 2019; Wang et al., 2024). Through this framing, the review aspires to furnish an analytical framework for bilingual aphasia research that diverges from traditional, tool-oriented syntheses, instead positioning the computational model as a controlled, in-silico experimental platform for examining how failures in cognitive control are represented, manipulated, and tested within a formalised system.

### 1.3. Research Objectives and Framework

To systematically structure the existing body of research and enhance the focus of the review inquiry, this review employs the SPIDER framework (Cooke et al., 2012) to delineate the Sample, Phenomenon of Interest, Design, and Research type, thereby guiding the overall process of literature retrieval, screening, and synthesis. Specifically, the Sample for this review is confined to studies that apply computational models to investigate the language processing systems of bilingual or multilingual aphasia. The Phenomenon of Interest centres on higher-order, bilingual-specific language deficits associated with failures in cross-language cognitive control mechanisms (Goral et al., 2019; Russell-Meill et al., 2025). At the Design level, this review critically examines how computational lesions are implemented within the included literature, how pathological phenomena are simulated on this basis, and how mechanistic interpretations are substantiated. The Research type is defined as a systematic review.

The objective of this review is not to propose a novel theoretical framework but rather to provide a structured synthesis, comparison, and critical appraisal of existing research. To this end, a dual-layer analytical framework integrating descriptive mapping and evaluative analysis is adopted. At the descriptive level, attention is directed toward the types of models employed, the technical approaches to computational lesioning, the phenomena simulated, and the distribution of studies across these dimensions. At the evaluative level, the review further examines the extent to which these studies furnish validation evidence in support of their mechanistic claims and explores how their findings interface with established cognitive neuroscience theories (Sajid et al., 2020; Walker & Hickok, 2016).

To strengthen the systematic nature and comparability of the analysis, two operational instruments were developed solely for the purpose of integrated synthesis within this review. These instruments were derived inductively from methodological features that recurred in the included literature during the data extraction phase. The first instrument comprises a five-dimensional classification scheme for encoding the implementation methods of computational lesions, grounded in the systematic extraction and categorisation of lesioning approaches identified across the 42 included publications. The second instrument consists of a hierarchical criterion for comparing the strength of evidence supporting mechanistic interpretations, established through a comprehensive analysis of extant validation methods. It should be noted that both instruments are employed exclusively to enhance the operationalisability and transparency of comparisons across diverse studies within this review and do not constitute independent theoretical frameworks proposed by this article (Nadeau, 2019; Sajid et al., 2021).

Through this analytical pathway, the present review aims to render a systematic appraisal of the current state of computational modelling research in bilingual aphasia across three dimensions: methodological distribution, depth of theoretical explanation, and potential for clinical translation. This appraisal further establishes the foundation for the subsequent discussion, wherein the core unresolved issues confronting the field are further distilled (Russell-Meill et al., 2025).

Before proceeding to the body of the review, a brief note on its genre structure may help prevent confusion of reader expectations. This paper performs three functions simultaneously, each localised in different sections. First, it is a systematic review conducted in accordance with the Preferred Reporting Items for Systematic Reviews and Meta-Analyses (PRISMA) 2020 (Section 2 and Section 3), reporting the systematic search, screening, coding, and quantitative and qualitative synthesis of 42 included publications. Second, it inductively develops, in the course of the review, three analytical instruments: the five-dimensional lesion coding scheme, the four-tier evidence hierarchy, and the seven-dimension methodological quality assessment, whose purpose is not to advance a new theoretical taxonomy but to provide a unified operational framework for cross-study comparison (Section 2.3). Third, it offers a conceptual synthesis and critical discussion on the foundation of the review findings (Section 4), assessing the current state of the field, its points of consensus and divergence, and its principal unresolved questions. These three functions are not separate papers but different analytical layers of a single review. The opening of each major section below indicates the analytical level and function it serves.

## 2. Methods

### 2.1. Review Design and Registration

To fulfil the normative requirements of a systematic review, the protocol for this study was prospectively registered with the International Prospective Register of Systematic Reviews (PROSPERO; ID: 1266436). The entire research process was conducted in strict accordance with the Preferred Reporting Items for Systematic Reviews and Meta-Analyses (PRISMA) 2020 guidelines to ensure methodological transparency, reproducibility, and reliability of the findings (Page et al., 2021; Tugwell & Tovey, 2021).

The core premise of this review is to conceptualise the computational model as a formalised experimental system wherein parameters, connections, and processing pathways can be precisely manipulated within a virtual environment, thereby serving as a controlled causal experimental platform for testing hypotheses concerning cognitive impairment. Traditional clinical behavioural research predominantly observes static post-injury outcomes or infers correlational relationships among variables, thereby having difficulty directly and systematically testing causal hypotheses. Specifically, the hypothesis that damage to a particular cognitive component leads to a specific behavioural pattern is difficult to test, given that such experiments are often precluded in patient populations due to ethical and practical constraints. Computational modelling, in contrast, permits the generation of testable causal predictions through the precise implementation of computational lesions within a formalised, in-silico system (Palminteri et al., 2017), thereby shifting the research focus from correlation toward the exploration of causal mechanisms. However, a critical caveat must be acknowledged: the mechanistic testing realised by a computational model is computational causality, contingent upon the model’s architecture and parametric assumptions, rather than being synonymous with physical causality at the neurobiological level. This distinction is of paramount importance for evaluating the explanatory power of models, particularly those characterised by opaque internal mechanisms often described as “black-box” architectures (McClelland, 2010; Xu et al., 2026). Notwithstanding this fundamental limitation, the computational modelling platform, by virtue of its high degree of controllability and reproducibility, offers a complementary avenue of investigation that is ethically and experimentally difficult to achieve within traditional clinical research. This review, therefore, evaluates not only the technical performance of models but also, more critically, their potential and limitations in elucidating the computational mechanisms underpinning the breakdown of bilingual cognitive control. Specifically, this article is concerned with the extent to which a model can function as a hypothesis-driven tool for mechanistic testing at the modelling level.

To align with this specific research orientation, the formulation of inclusion and exclusion criteria was guided by the following core principle: a deliberate distinction was drawn between mechanism-oriented and application-oriented computational studies, with the analytical focus directed toward the former. This approach served to enhance the internal coherence between the review sample and the research question. The inclusion criteria were as follows: (1) the sample population focused on the language processing system of bilingual or multilingual individuals with aphasia, encompassing clinical studies and corpus analyses involving actual patients, as well as exploratory computational work aimed at simulating such systems. (2) The research type was restricted to studies intended for mechanistic simulation, defined as original investigations aimed at probing or explaining the intrinsic cognitive-computational mechanisms underlying bilingual aphasia-specific language deficits. (3) The research content necessitated the application of a computational model to simulate specific language functional deficits associated with bilingual aphasia. (4) The core research method had to involve the application of a computational model to simulate, dissect, or intervene in specific language deficits related to bilingual aphasia. (5) The results were required to report concrete findings pertaining to mechanistic explanation, behavioural prediction, or theoretical testing derived from the simulation method. The exclusion criteria were as follows: (1) studies that exclusively addressed monolingual aphasia or did not involve cross-language interaction mechanisms. (2) Editorials, commentaries, conference abstracts, study protocols that merely outlined research plans without presenting results, and generic methodological guidelines. (3) Technical literature that focused solely on general engineering performance metrics without integrating the specific language-cognitive characteristics of aphasic patients. (4) Studies that, while involving bilingual aphasia, reported only on quality-of-life scales or psychosocial factors and lacked core language behavioural data essential for model parameter calibration. (5) Investigations confined to traditional behavioural task assessments without explicit computational parameterisation (Cooke et al., 2012).

It should be noted that the distinction between mechanism-oriented and application-oriented studies is employed here as a heuristic device for focusing the review’s analytical lens, rather than as a strict ontological claim about the nature of individual studies. Some investigations may combine mechanistic explanatory intent with application-oriented predictive goals; the categorisation adopted in this review reflects the analytical priority assigned during screening and is not intended as an exclusive judgment on a study’s overall contribution.

It should be noted that the above criteria define eligibility for the core mechanistic simulation sample (*n* = 12), to which the five-dimensional lesion coding scheme (Section 2.3.1) and the four-tier evidence hierarchy (Section 2.3.2) were applied. Recognising that the interpretation of computational models in bilingual aphasia requires both clinical contextualisation and methodological framing, this review additionally incorporated two further categories of literature under a layered inclusion design. Empirical and clinical studies (*n* = 19) were retained when they provided direct behavioural or neuropsychological evidence on bilingual aphasia, including code-switching patterns, treatment outcomes, and language control assessments, which serve as the empirical referents against which computational models are developed and validated. Review and methodology papers (*n* = 11) were retained when they offered systematic synthesis of treatment evidence, methodological guidance for computational modelling, or theoretical frameworks for bilingual language control. These two categories were not subjected to lesion coding or tier classification (coded as not applicable; see Table S1) but contributed to the methodological quality assessment (Section 2.3.3) and the integrated discussion of clinical translation (Section 3.3). Two studies warrant explicit justification. S4 (Peñaloza et al., 2020) is a registered trial protocol for the PROCoM randomised controlled trial. Although study protocols without results were nominally excluded under criterion (2), S4 was retained because it provides the most detailed published account of the BiLex model’s personalised calibration methodology, information that is directly relevant to interpreting the lesion implementation approach in related simulation studies (S2, S5, S30). S27 (Walker & Hickok, 2016) presents the SLAM model, which addresses monolingual rather than bilingual aphasia and would therefore fall under exclusion criterion (1). It was retained because SLAM represents a direct computational precursor to the bilingual BiLex model and exemplifies the parameter-level lesion methodology within the broader computational-neuropsychological tradition (Dell et al., 1997; Foygel & Dell, 2000), thereby providing essential methodological context. Both exceptions are flagged in Table S1.

Through the application of the aforementioned criteria and the layered inclusion design, this review identified a corpus of 42 publications that, while heterogeneous in study type, spanning computational simulation studies, empirical clinical investigations, and review or methodology papers, is collectively oriented toward understanding the role of computational approaches in bilingual aphasia research. Each of the three categories serves a distinct analytical function within the overall synthesis: the 12 simulation studies constitute the core sample for mechanistic analysis, the 19 empirical studies supply the clinical evidence base, and the 11 review or methodology papers furnish theoretical and methodological framing.

### 2.2. Literature Search Strategy

**Figure 1:**
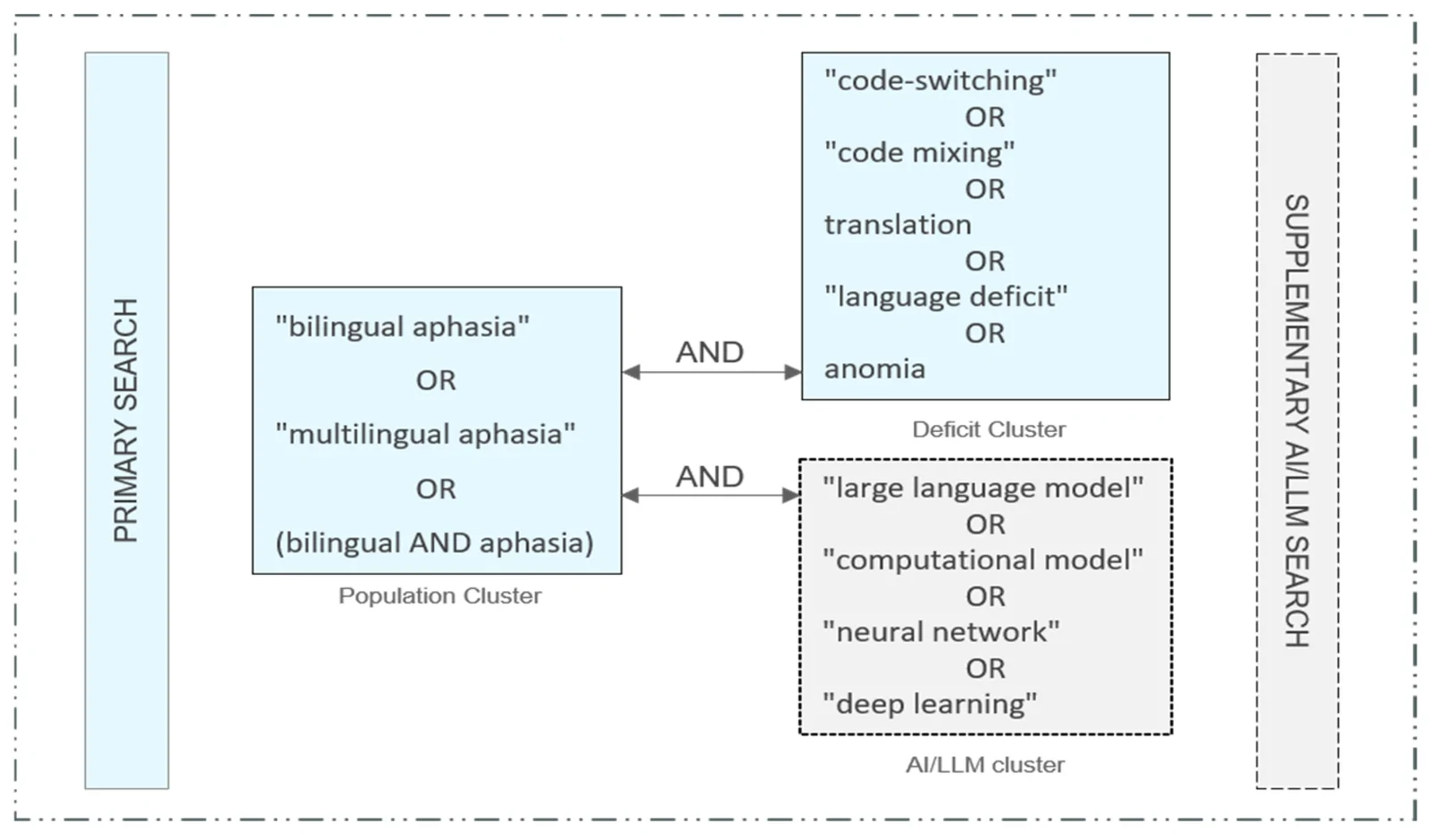
Schematic diagram of the Boolean search logic. Note: The upper panel illustrates the primary search, combining the population cluster with the deficit cluster via the AND operator. The lower panel illustrates the supplementary AI/LLM search, combining the same population cluster with a cluster of artificial intelligence and large language model terms. Terms within each cluster were connected by OR. Blue solid lines/outlines denote the primary search, and grey dotted lines/outlines denote the supplementary AI/LLM search.

To enhance the comprehensiveness and reproducibility of the search process, a systematic retrieval protocol was developed for this review. The primary search strategy was constructed around two principal conceptual clusters: target population/disorder and specific language deficits. Computational model-related terms were deliberately excluded from the primary search to maximise sensitivity; the identification of studies involving computational approaches was instead performed during the title, abstract, and full-text screening stages. As depicted in Figure 1, core search terms within each cluster were combined using the Boolean operator OR, and the two clusters were connected by AND. Recognising that computationally focused studies may not be indexed under the specific deficit terms employed in the primary search, a supplementary search was conducted combining the population cluster with a dedicated set of artificial intelligence and large language model terms, as illustrated in the lower panel of Figure 1. Both the primary and supplementary searches were executed across three databases: Web of Science, PubMed, and IEEE Xplore. The design and adaptation of the search strategy were guided by the following principles: (1) no restriction was placed on the start date of publication, with the search conducted up to the date of execution, 19 January 2026; (2) eligible publication types included peer-reviewed journal articles, conference papers, and publicly available preprints, while book chapters, editorials, commentaries, and dissertations were excluded; (3) the language was restricted to English; and (4) citation tracking was additionally employed as a supplementary method. It is pertinent to acknowledge that restricting the search language to English for reasons of feasibility and consistency may introduce geographical bias, a limitation that is recognised in the Section 4 (Moher et al., 2009; Page et al., 2021). The complete search strings, adapted to the specific syntax of each database, are presented in Table 1. The Boolean logic of the search strategy is illustrated in Figure 1. The primary search deliberately excluded model-specific technical terms in order to maximise sensitivity: given the inconsistency with which computational modelling studies in this domain are indexed, restricting the primary search to deficit-specific terms allowed the retrieval of relevant work regardless of the terminological conventions adopted by individual authors. The supplementary AI/LLM search partially compensated for this deliberate exclusion, and the citation tracking procedure provided an additional safeguard against omission. It is nevertheless acknowledged that some studies employing computational methods under narrower technical labels may have been missed, and this constitutes a limitation of the search strategy.

Recognising that research output in the computational modelling domain may be dispersed across multiple disciplines and that emerging studies often debut as conference papers, a supplementary strategy of citation-based snowballing was introduced beyond the primary database searches to mitigate publication bias and capture significant literature potentially missed by conventional retrieval methods (Wohlin, 2014). Specifically, two representative publications were selected as seed papers: one was the seminal work by Sajid et al. (2020) published in 2020, which represented an early systematic application of computational neural models to simulate bilingual naming deficits; the other was a recent review by Wang et al. (2024) published in 2024, encompassing the themes of computational modelling and bilingual aphasia. The selection of these two references was intended to balance foundational grounding with a forward-looking perspective on the field. Iterative tracking was conducted using the Litmaps tool (Litmaps, Wellington, New Zealand) until successive iterations yielded no further literature meeting the inclusion criteria. It must be emphasised that this method served strictly as a supplement, and all literature identified through this pathway was subjected to the identical rigorous screening process applied to the primary search records. Preprints were considered eligible for inclusion given the rapid publication cycle of computational modelling research, in which substantive findings frequently appear on preprint servers prior to formal peer review and may take months or longer to appear in final published form. Dissertations and book chapters were excluded: The former are not uniformly subject to peer review, and the latter typically present synthesised rather than original empirical findings, limiting their suitability for the detailed methodological extraction required by this review.

**Figure 2:**
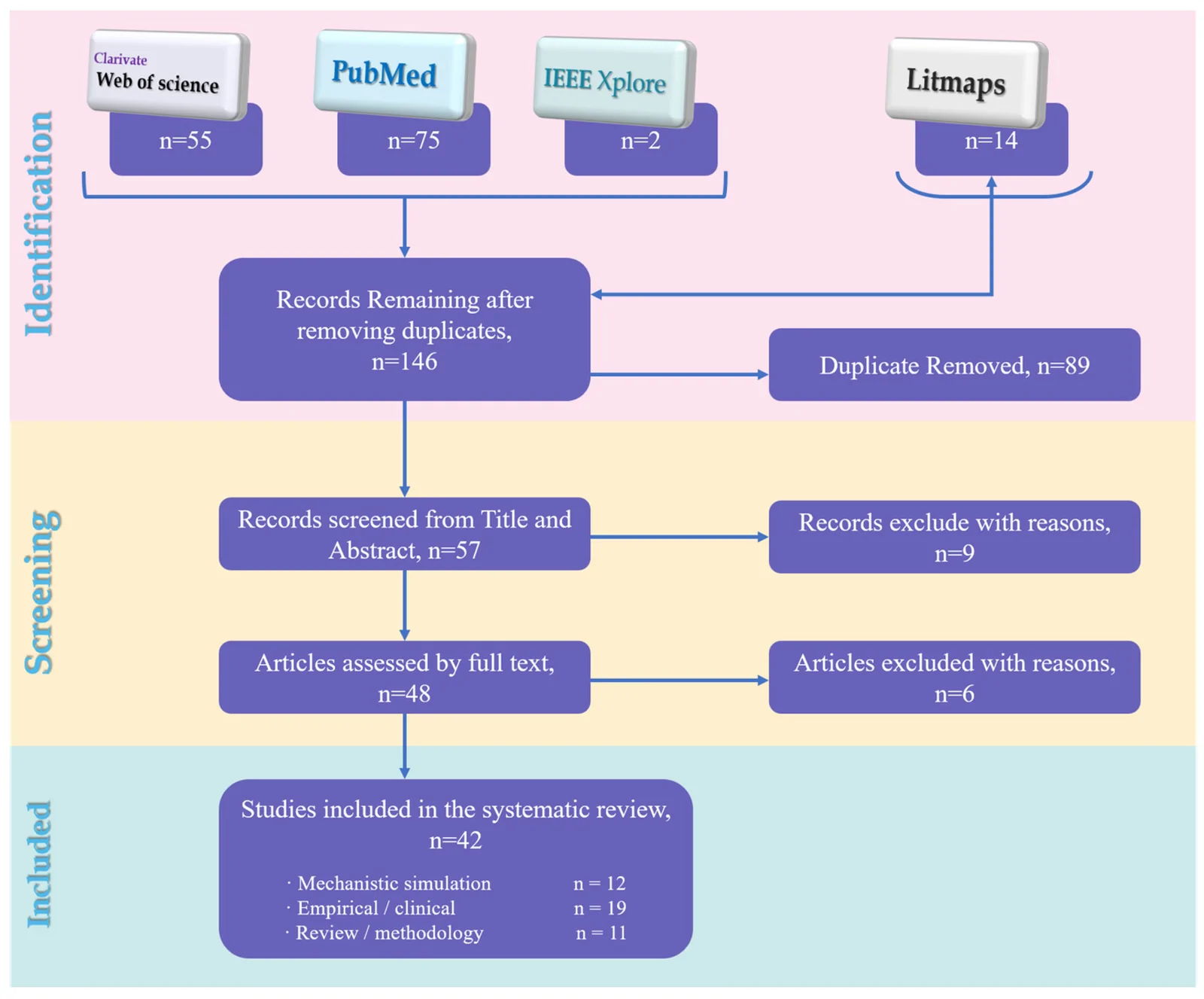
PRISMA flow diagram of the study selection process.

The entire literature screening process strictly adhered to the standardised procedures outlined in the PRISMA flow diagram (Figure 2) (Tranfield et al., 2003). As illustrated, the initial search yielded a total of 146 records, with all databases queried on 19 January 2026 using the advanced search strings detailed in Table 1. Following the application of Zotero’s automated deduplication function (Zotero, Corporation for Digital Scholarship, Vienna, VA, USA) supplemented by manual verification, 57 unique records were obtained. The high duplication rate was primarily attributable to substantial overlap in journal coverage across the queried databases, particularly within biomedical and interdisciplinary fields, coupled with a deliberately broad search strategy designed to maximise sensitivity, resulting in the retrieval and inclusion of the same publication across multiple databases. However, not all of these records fell within the scope of interest for this review. For instance, studies that solely focused on behavioural task performance without incorporating an explicit computational parameterisation process were excluded. Initial screening of titles and abstracts led to the exclusion of nine records deemed off-topic, with content primarily centred on neuroimaging, meta-analyses, or traditional clinical behavioural experiments that did not employ any parameterised computational system for simulation or analysis. Subsequently, the remaining 48 publications underwent full-text review, a stage at which rigorous assessment was conducted against the predefined inclusion and exclusion criteria. This process resulted in the further exclusion of six publications, primarily because their core focus was on discussing the general relationship between executive function and conversational strategies, or because they solely reported clinical evidence of rehabilitation efficacy without incorporating computational models as a central component of their methodology. For example, two of these publications were excluded on the grounds that they did not demonstrate direct applicability to bilingual aphasia. Following this multi-stage screening, a final corpus of 42 publications was confirmed as the core analytical sample for this systematic review. Additionally, during the screening process, another 16 records were categorised as application-oriented studies. Although these publications are not incorporated into the quantitative or qualitative syntheses of the core sample, their existence and relevance will be invoked in the Section 4 to help delineate the broader landscape of the field and the current state of the mechanism-application interface.

### 2.3. Data Extraction and Coding Framework

To transform the included publications into structured data amenable to analysis, a standardised coding form was initially designed for this review. The first author coded all 42 publications. To assess the reliability of the coding instruments, a second author independently coded a randomly selected subset of 10 publications (approximately 24% of the corpus) using the same coding frameworks described in Section 2.3.1, Section 2.3.2 and Section 2.3.3, without access to the first author’s codes. Inter-rater reliability was assessed using Cohen’s *κ* for each instrument. Agreement was perfect for the five-dimensional lesion coding scheme (*κ* = 1.00, raw agreement 100%) and for the four-tier evidence hierarchy classification (*κ* = 1.00, raw agreement 100%). For the seven-dimension methodological quality assessment, agreement was substantial (*κ* = 0.65, weighted *κ* = 0.70, raw agreement 83%); all 14 disagreements were between adjacent categories (e.g., High vs. Medium), with no extreme disagreements (High vs. Low). Discrepancies between coders were resolved through discussion, and the resulting consensus codes were used for all subsequent analyses. The complete per-study codes are reported in Table S1. Where a study’s lesion implementation could reasonably be assigned to more than one dimension, both were recorded and the rationale noted. Such ambiguity occurred most commonly at the boundary between parameter-level and control-level lesions, where the distinction between adjusting inhibitory strength (D1) and weakening gating units (D3) depends on the level of abstraction at which the model is described. The in-depth analysis presented in this review is principally anchored in the following two operational instruments, which were inductively derived in response to the specific research questions.

#### 2.3.1. A Five-Dimensional Coding Scheme for Implementing Computational Lesions

To avoid an overly generic application of the “computational lesion” concept and to enhance the operationalizability of comparisons across disparate studies, this review proposes a five-dimensional coding scheme for characterising the implementation methods of computational lesions, derived from the synthesis of the included literature and presented in Table 2. This scheme is grounded in the systematic extraction of methodological features from the included publications. It adopts a broad definition of computational lesion, encompassing any approach whereby researchers manipulate a model’s internal parameters, knowledge representations, structural components, calibration processes, or external input conditions to simulate functionally constrained states of a cognitive system. Recognising that multiple intervention methods are frequently employed concurrently within actual research, this framework permits multi-label coding of individual publications.

Specifically, the first dimension, “parameter-level lesion”, refers to direct alterations of a model’s internal parameters, such as connection weights, activation thresholds, inhibitory strength, learning rate, or noise levels, in order to simulate impaired states, such as diminished processing efficiency, unstable activation, or weakened control capacity. The second dimension, Representational-level lesion, involves interventions targeting the model’s internal knowledge representations themselves; examples include attenuating certain semantic features, deleting specific lexical entries, or reducing the distinctiveness of cross-language mappings, thereby simulating loss, degradation, or confusion at the representational level. The third dimension, Control-level lesion, pertains primarily to interventions on core control processes such as language selection, switching, inhibition, or monitoring. This includes weakening gating units, increasing task-switching costs, or disrupting goal-language schema signals to simulate bilingual control deficits arising from damage to prefrontal-subcortical control networks. The fourth dimension, Architectural-level lesion, denotes alterations to the structural components of the model, including the removal, attenuation, or substitution of specific functional modules, or the adjustment of connective pathways between modules, to simulate system damage with greater structural specificity. The fifth dimension, Input/Environmental-level lesion, refers to the induction of functional anomalies by modifying the external stimuli, task demands, or linguistic environment received by the model. Examples include restricting input information, manipulating language cues, or increasing switching load, thereby examining changes in model performance under constrained conditions.

The purpose of this five-dimensional coding scheme is to furnish a unified descriptive tool for facilitating cross-sectional comparisons within this review, rather than to advance a novel theoretical taxonomy. By means of this scheme, the review is better positioned to identify the predominant preferences of different model types in implementing computational lesions and to ascertain whether stable correspondences exist between these technical pathways and the types of deficits being simulated.

#### 2.3.2. Hierarchical Criteria for Strength of Mechanistic Evidence

To guard against conflating any form of model fitting with mechanistic explanation, this review adopts a set of hierarchical criteria for evaluating the strength of evidence presented in the included literature. The objective is to determine the extent to which a study supports mechanistic hypothesis testing at the modelling level. In this context, “mechanistic explanation” signifies that the literature not only demonstrates the model’s capacity to reproduce a particular behavioural outcome but, more critically, establishes a stable and testable correspondence between a specific class of lesion operation and a specific class of behavioural anomaly through controlled manipulation of the model’s internal components or processing pathways.

Drawing upon established principles in causal modelling and model validation, this review condenses the relevant evidence into four operational criteria, against which the literature is stratified. The first criterion is independent validation: the key predictions generated by the model on one dataset must be corroborated by a separate, independent dataset, which may derive from distinct patient samples, different task paradigms, or alternative research contexts. The second criterion is differentiable prediction: the model must be capable of generating mutually distinguishable behavioural predictions for different lesion patterns, and these predictions must exhibit a qualitative or quantitative correspondence with distinct clinical subtypes or lesion profiles. The third criterion is quantitative goodness-of-fit: the study must report the fit between model output and actual patient data and demonstrate that the model significantly outperforms a reasonable baseline model, rather than relying solely on superficial similarity. The fourth criterion is specificity testing: through the inclusion of control lesion operations, the study must demonstrate that the target behavioural deficit is not merely a consequence of any generic impairment but is more strongly associated with a specific lesion pattern.

In terms of specific stratification, this review classifies publications failing to meet any of the above criteria as Tier 0, considered as descriptive fitting or hypothesis generation. Publications meeting any single criterion are classified as Tier 1, regarded as weak suggestive evidence. Publications meeting any two criteria, provided that at least one of them is differentiable prediction or specificity testing, are classified as Tier 2, deemed as moderate indicative evidence. Publications meeting three or more criteria are classified as Tier 3, considered as strong supportive evidence. It is imperative to emphasise that this hierarchical system constitutes a working evaluative framework synthesised by this review to facilitate methodological comparison and critical discussion; its primary utility lies in enabling a relative and circumspect comparison of the explanatory power of the existing literature.

To ensure consistency in the application of these criteria, the following decision rules were adopted for cases falling near category boundaries. A study that reports a quantitative fit metric without a baseline comparison model is coded as not meeting C3 (quantitative goodness-of-fit), on the grounds that the metric alone, in the absence of a comparative benchmark, does not establish that the model has outperformed a simpler or null model. A study that compares lesion conditions but does not include a control lesion operation specifically designed to test the necessity of the target lesion pattern is coded as not meeting C4 (specificity testing). A study whose independent validation is conducted on a held-out subset of the same dataset, rather than an independent sample, is coded as not meeting C1; the held-out subset approach, while methodologically preferable to within-sample fitting, does not constitute independent validation as operationalised in this framework. A study that simulates only one lesion pattern or compares a lesioned model solely to an intact baseline, without comparing behavioural predictions across at least two distinct lesion configurations, is coded as not meeting C2 (differentiable prediction), on the grounds that differentiable prediction requires the model to generate distinguishable output patterns for different lesion types. All borderline coding decisions were discussed between coders during the consensus resolution process, and the rationale for each decision is recorded in Table S1.

#### 2.3.3. Methodological Quality Assessment

It should be noted that this quality assessment evaluates methodological transparency and rigour rather than theoretical alignment with any particular modelling paradigm. A study rooted in a connectionist framework and a study employing a deep learning architecture are evaluated against the same methodological standards, irrespective of whether the review authors regard one theoretical approach as more conducive to mechanistic explanation than the other. This methodological agnosticism is essential to the review design, but it entails an asymmetry that should be borne in mind when interpreting the results: models designed for interpretability may appear to perform better on dimensions such as theoretical justification (Q3) or contextualisation (Q7) than models optimised primarily for predictive accuracy, and this difference reflects the epistemic priorities embedded in the assessment dimensions rather than an inherent superiority of one modelling paradigm over another.

Furthermore, a supplementary methodological quality assessment was conducted on all publications included in the core sample. This assessment addresses the rigour and transparency of study execution, encompassing seven principal dimensions: transparency and reproducibility of model description; accessibility of code and data; theoretical or empirical justification for the lesioning method; ecological validity of the data utilised; appropriateness of the evaluation methods; rigour of result interpretation; and depth of clinical or theoretical contextualisation. Each dimension is rated on a three-point scale (High/Medium/Low). This assessment framework draws upon core principles articulated in systematic review reporting guidelines (the PRISMA Statement) (Moher et al., 2009) and is intended to provide background information concerning the methodological maturity of the field for the subsequent integrated discussion, rather than serving as a criterion for exclusion.

### 2.4. Integrated Analytical Approach

Finally, this review employs a mixed-methods approach, integrating both quantitative and qualitative analyses to synthesise the extracted data. The quantitative analysis will primarily utilise descriptive statistics to delineate overarching trends and characteristics within the literature corpus, encompassing dimensions such as temporal and geographical distribution, model typologies, foci of simulated deficits, distribution of lesion implementation techniques, and the stratification of evidence strength across tiers. In parallel, the qualitative analysis will adopt a thematic synthesis methodology. Guided by the overarching theme of theory-driven mechanistic simulation, this approach will undertake an in-depth comparison of the explanatory logic underpinning divergent technical pathways. Furthermore, drawing upon the two core analytical frameworks articulated previously, namely, the five-dimensional coding scheme for computational lesions and the hierarchical criteria for mechanistic evidence, this analysis will interrogate the progress achieved, the consensuses established, and the core bottlenecks confronted by the current literature in its pursuit of the dual objectives of testing causal mechanisms and generating clinical and theoretical insights.

## 3. Results

For the purposes of this review, a “computational model” is defined inclusively as any computational system that, through adjustable parameters and algorithmic structures, is purposefully designed to simulate, explain, or predict language pathology phenomena in bilingual aphasia. This encompasses classical computational neural models, contemporary language models, and traditional machine learning models. As discussed in Section 1.2, the literature subsumed under this definition spans epistemologically distinct categories; the review’s three analytical instruments (Section 2.3.1, Section 2.3.2 and Section 2.3.3), namely, the five-dimensional lesion coding scheme, the four-tier evidence hierarchy, and the seven-dimension methodological quality assessment, are designed to enable systematic differentiation across these categories within an inclusively constituted corpus. The 42 publications included for analysis thus constitute a heterogeneous corpus, collectively spanning foundational theoretical modelling, computational analysis of clinical phenomena, and treatment response prediction. They are unified not by a shared technical architecture but by a common reliance on parameterised computational systems to formally characterise the language control deficits observed in bilingual aphasia. It is upon this common baseline that the subsequent analysis of divergent technical pathways, simulation efficacy, and inherent limitations is conducted.

### 3.1. Overview of Included Studies

Following systematic screening, a total of 42 publications constitute the core analytical sample for this review, with key information summarised in Table S1 (see Supplementary Materials). These publications span the period from 2010 to 2025 and exhibit broad geographical distribution, encompassing research conducted across North America, Europe, Asia, and other regions. Study designs range from rigorously controlled clinical empirical investigations to in-depth mechanistic explorations via computational simulation. Sample sizes vary from single cases to cohorts comprising several dozen patients, and the participant populations involve diverse bilingual or multilingual pairings, including English–Spanish and Chinese–English. Notably, only one study, designated S29, employed a large pre-trained language model as its core methodology (Bao et al., 2025).

**Figure 3:**
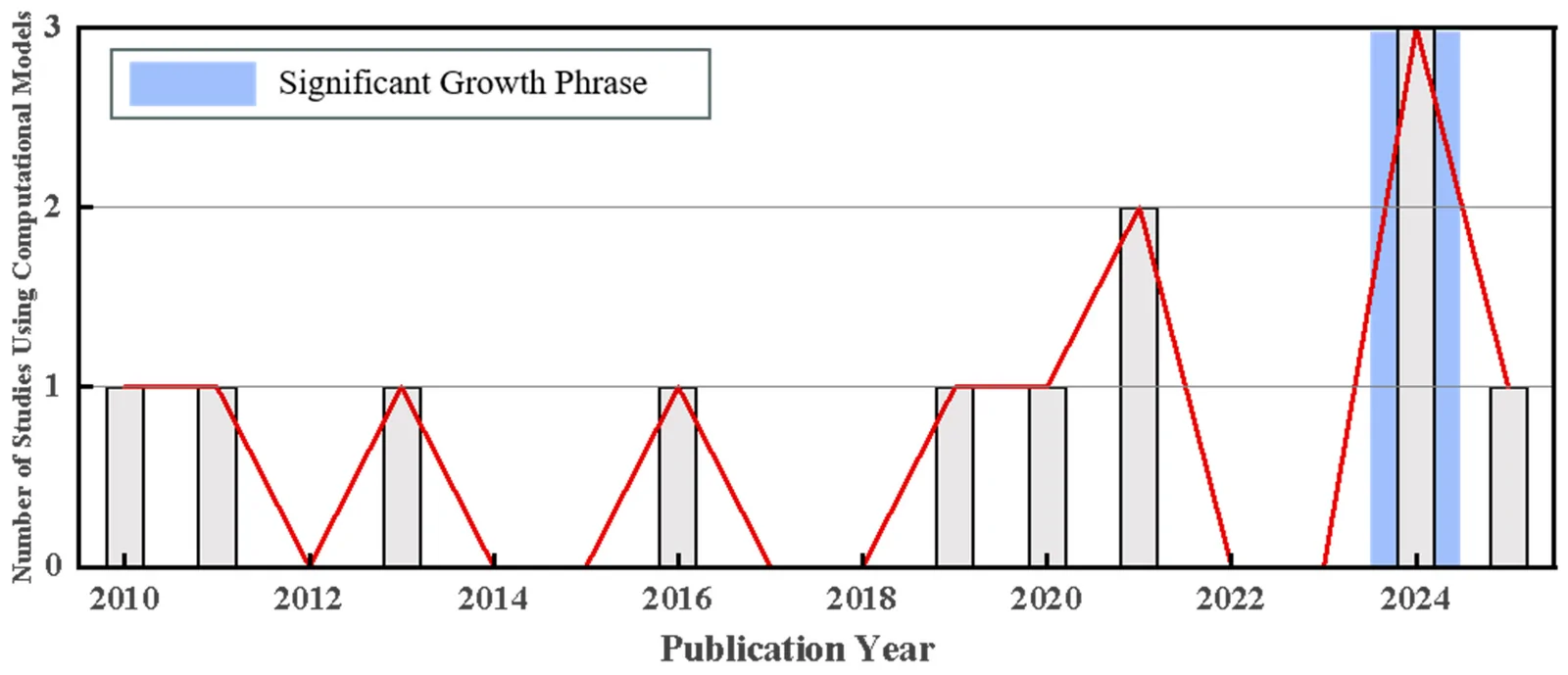
Annual publication trend of computational modelling studies (2010–2025). The grey bars indicate the annual number of studies employing computational models, the red line shows the overall publication trend, and the blue shaded band highlights the significant growth phase, in which the number of publications reached an all-time high in 2024.

In terms of temporal evolution, the annual distribution of the 12 core computational simulation studies exhibits a fluctuating upward trend, as illustrated in Figure 3. Early contributions appeared sporadically, with one study each in 2010 and 2011, one in 2013, and an intermittent but sustained output from 2016 to 2021, with one to two studies appearing in most years. An increase was observed in 2024, which saw the publication of three computational simulation studies, the highest annual count recorded, and one new study has already appeared in 2025. This trend suggests that the research trajectory of employing computational models to simulate language deficits in bilingual aphasia is garnering sustained interest, albeit the absolute number of studies remains modest.

An analysis of geographical and linguistic backgrounds reveals that approximately seventy percent of the studies were conducted within North American and Western European academic settings. The core language pairs investigated are predominantly English–Spanish, with considerably fewer studies addressing language pairs such as Chinese or Arabic. This concentration of linguistic backgrounds may constrain a universal understanding of the mechanisms underlying brain damage in bilinguals.

**Figure 4:**
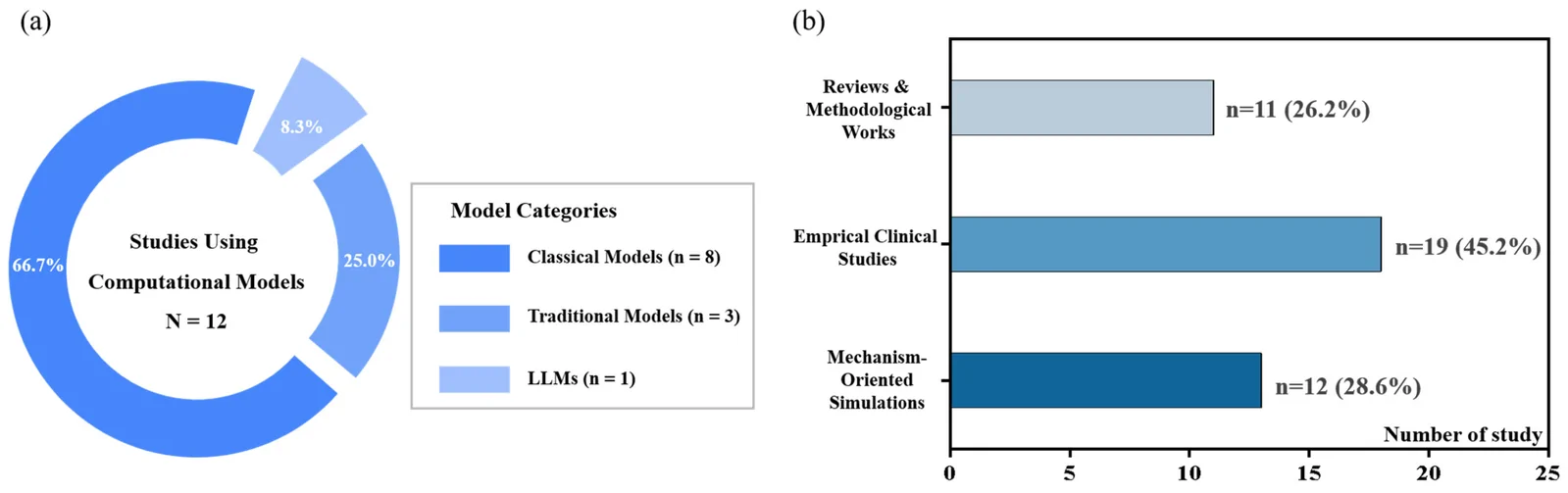
Distribution of core model architectures and study designs: (**a**) Distribution of core computational model architectures. Based on the 12 computational simulation studies centred on mechanistic simulation, the proportions of different model architectures are presented. (**b**) Distribution of primary study designs within the included literature. Based on all 42 included publications, studies are categorised by design type.

Analysis of the 12 computational simulation studies, as depicted in Figure 4a, reveals that the field remains dominated by classical models grounded in explicit cognitive theory and purpose-built for simulating language processing mechanisms, accounting for eight studies (66.7%). In contrast, traditional machine learning algorithms oriented toward classification or prediction constitute three studies (25.0%), while the application of general-purpose LLMs pre-trained on massive corpora is minimal, represented by only a single study (8.3%). This distribution indicates that despite rapid advances in AI technology, specialised models capable of elucidating complex mechanisms of bilingual control and impairment remain central to current research. This pattern implies that for a highly intricate neurocognitive disorder such as bilingual aphasia, data-driven prediction has not supplanted mechanism simulation grounded in cognitive theory.

With respect to the overall composition of the 42 studies, as shown in Figure 4b, empirical investigations based on actual clinical patient data account for 19 publications (45.2%), reflecting the field’s clinical orientation. Computational simulation studies specifically aimed at modelling language deficits represent 12 publications (28.6%), playing a pivotal role in bridging theory and experimentation. Additionally, review articles, meta-analyses, and methodological discussions constitute 11 publications (26.2%) (Adikari et al., 2024; Ansaldo & Saidi, 2014; Gusenbauer & Haddaway, 2020; Kong et al., 2014, 2016; S. Lee & Faroqi-Shah, 2024; Moher et al., 2009; Nadeau, 2019; Russell-Meill et al., 2025; Senadheera et al., 2024; Wang et al., 2024; Zhong, 2024), indicating that the academic community is actively engaged in systematic consolidation and critical reflection upon the rapidly accumulating body of knowledge.

### 3.2. Spectrum of Computational Models

#### 3.2.1. Mechanistic Simulation of Language Deficits

**Figure 5:**
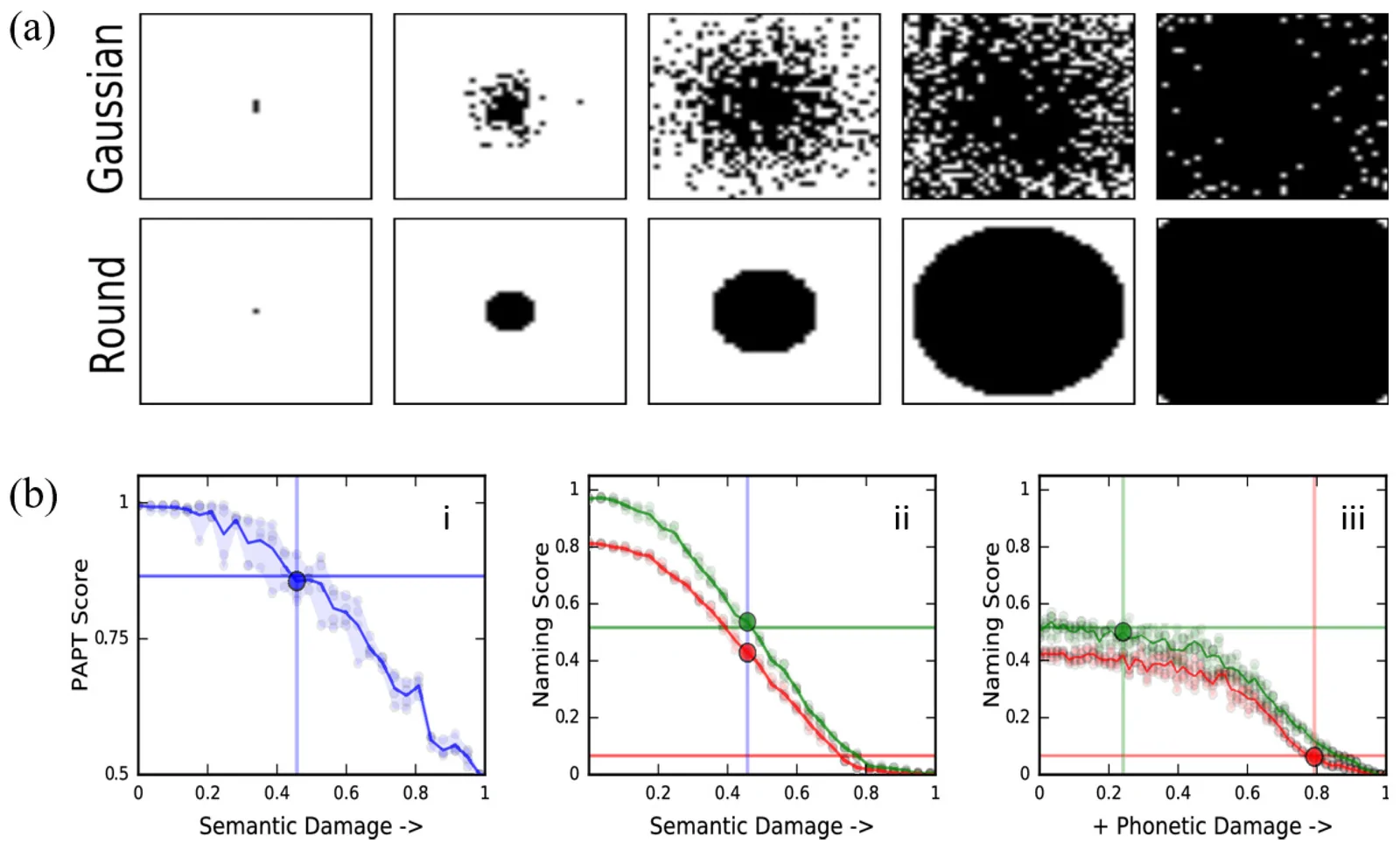
Schematic of the BiLex model for simulating lexical retrieval deficits in bilingual aphasia: (**a**) Lesion simulation. Focal brain injury is simulated via the deletion of neurons (node/connection removal) within specific regions, with lesion severity adjustable from mild (left) to severe (right) (Grasemann et al., 2021). (**b**) Data fitting process. Data fitting process. By systematically adjusting lesion parameters, the model’s semantic scores and bilingual naming accuracy are matched to the clinical data of actual patients (Grasemann et al., 2021). The sub-panels show: (**i**) the decrease in the model’s semantic performance on the Pyramids and Palm Trees test (PAPT) as semantic-map lesion damage increases, with the vertical line indicating the best-fitting damage and the horizontal line the patient’s target score; (**ii**) the effect of semantic damage on naming performance in English (green) and Spanish (red); and (**iii**) the additional lesion damage applied to the English and Spanish maps to match naming performance in both languages, where horizontal lines are the patient’s actual scores and vertical lines the best-fit lesion amounts.

Among the 42 included publications, a subset of 12 computational modelling studies was identified as having the explicit objective of mechanistically simulating specific language deficits. Their principal technical pathways converge upon the exploration of mechanisms underlying three distinct categories of deficit. The first category pertains to lexical retrieval and naming deficits. This constitutes the most prevalent modelling target, manifested by patients’ inability to accurately access or produce target words in tasks such as picture naming or word-picture matching. This deficit has emerged as a foundational entry point for modelling due to its implication of the competitive selection processes inherent in bilingual lexical representations, thereby rendering it relatively tractable for computational simulation. At the level of core mechanism simulation, the architectures of such models typically comprise a semantic layer, a bilingual lexical layer (L1, L2), and a phonological layer, as exemplified by the BiLex family of models in S2, S5, and S30 (Grasemann et al., 2011, 2021; Peñaloza et al., 2019), and connectionist models in S1 (Kiran et al., 2013a), and the SLAM model in S27 (Walker & Hickok, 2016). Figure 5a delineates the computational lesion operations; and Figure 5b explains model output and validation. The core mechanism simulated is the process of competition and selection between bilingual lexical representations. Computational lesion operations encompass the following four levels (Grasemann et al., 2010, 2011; Peñaloza et al., 2019). Parameter-level lesion involves systematically weakening or severing connection weights from the semantic layer to the lexical layer of a specific language to simulate impaired semantic access; attenuating connection weights from lexical nodes to the phonological layer to simulate lexical-phonological mapping deficits; or increasing the inhibitory connection strength from L1 to L2 within the lexical layer to simulate retrieval difficulties arising from heightened cross-language interference. Noise injection entails the introduction of Gaussian noise during the lexical layer activation computation process to simulate activation instability resulting from increased neural system noise. Node/connection removal involves the complete ablation of specific semantic feature nodes or lexical nodes to simulate the loss of particular categories or language-specific vocabulary attributable to focal brain injury. Regarding model output and validation, the model’s performance on naming tasks is fitted and compared against actual clinical behavioural data from patients.

**Figure 6:**
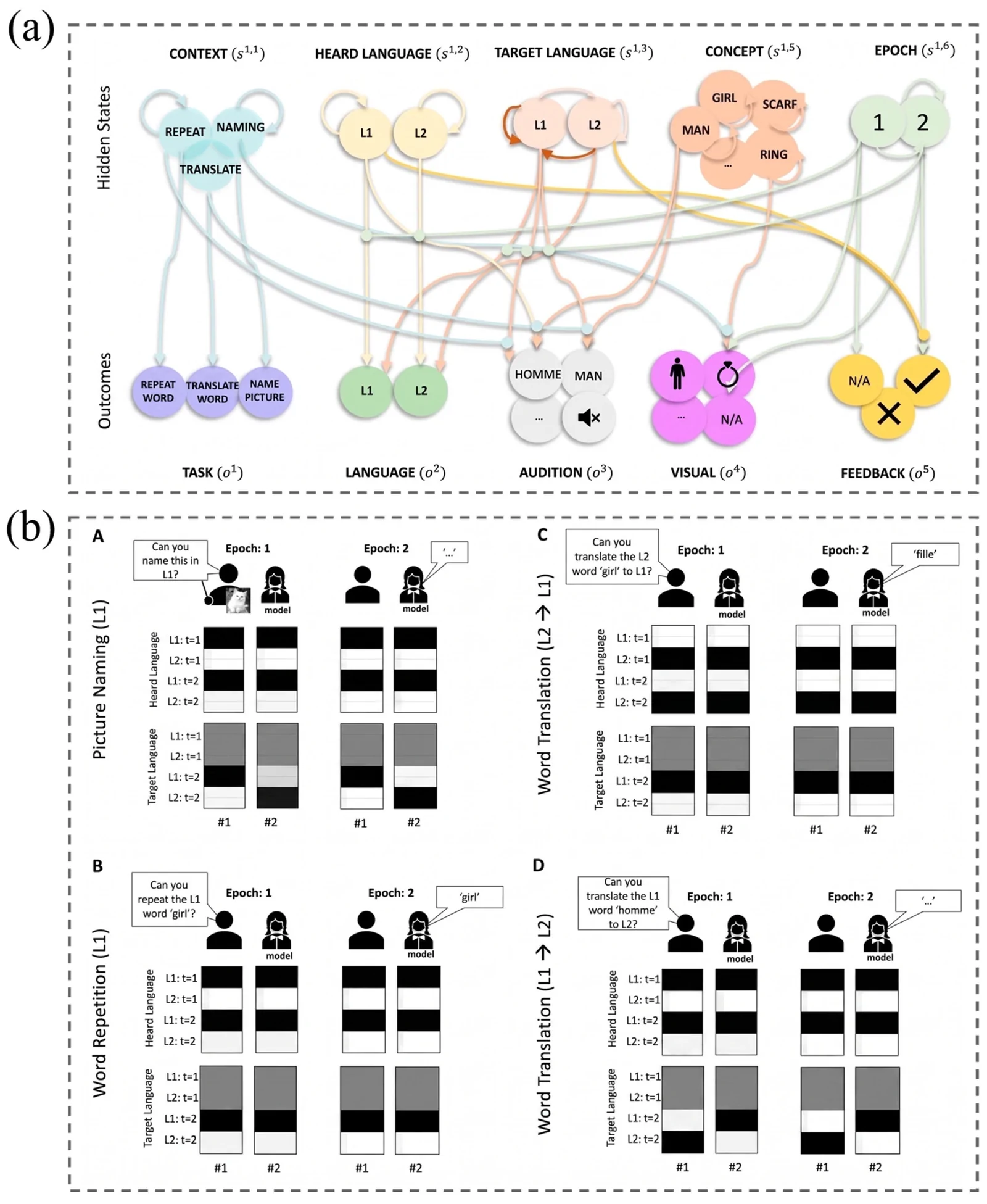
Schematic of the active inference model for simulating language control and code-switching deficits: (**a**) Model architecture. The diagram illustrates how control nodes, such as context and target language, exert top-down modulation over language selection (Sajid et al., 2020). (**b**) Belief updating process. By contrasting confidence changes, represented by greyscale intensity, between control and lesioned groups during language tasks, the model simulates code-switching deficits arising from weakened inhibition or attenuated control signals (Sajid et al., 2020). In panel (**a**), colours highlight the likelihood mappings and state-transition probabilities that are conserved across state factors and outcome modalities. In panel (**b**), the sub-panels show belief updating for (**A**) picture naming in L1, (**B**) word repetition in L1, (**C**) word translation from L2 to L1, and (**D**) word translation from L1 to L2; white represents an expected probability of zero, black a probability of one, and grey the intermediate gradations.

The second category concerns language control and code-switching deficits. Models in this category focus on simulating patients’ compromised ability to effectively manage and switch between languages. The core issue is impaired code-switching control, manifested as an inability to suppress lexical or grammatical structures of the non-target language in contexts where such intrusions are neither required nor appropriate, resulting in uncontrolled language mixing. Relevant studies include the active inference model in S7 (Sajid et al., 2020). Figure 6a presents the architecture of the generative model underlying the active inference framework. In terms of core mechanism simulation, such models augment a foundational language representation network with language task or control nodes. These nodes are responsible for the top-down modulation of target language activation thresholds or the inhibition of the non-target language contingent upon the current context. Figure 6b illustrates belief updating and language selection, thereby explicating the mechanisms of diminished inhibition and elevated switching costs. Computational lesion operations include the following five levels. Control signal attenuation involves weakening the connections from contextual input to the language task node or from the task node to language tags at the lexical layer. Reduced inhibitory function entails decreasing the inhibitory strength exerted by the language task node upon the non-target language. Control node lesion involves directly impairing the language task node itself. Elevated switching cost is implemented by increasing the computational cost associated with resetting the control state during a language switch. With respect to model output and validation, the code-switching frequency, error rates at switch points, switch types, and reaction time delays produced by the model during language switching tasks are compared against patient corpus data.

**Figure 7:**
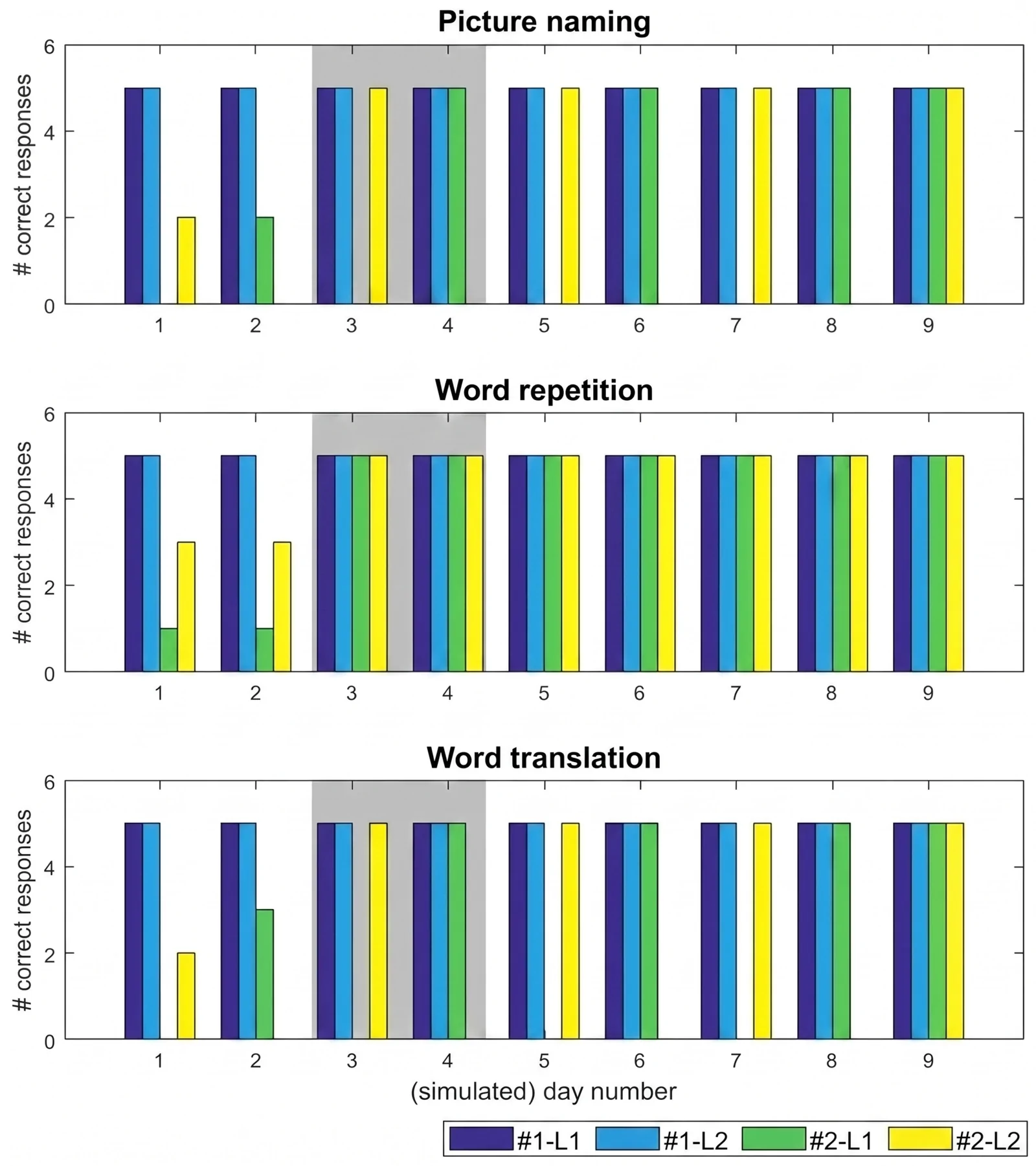
Simulated behavioural performance of the active inference model for translation deficits and recovery trajectories: Behavioural performance of the control group (#1) and the lesioned group (#2) over nine simulated days. The grey shaded area for Days 3 to 4 highlights asymmetric impairment in the translation task, such as intact L2 to L1 translation alongside impaired L1 → L2 translation, and alternating fluctuations in language dominance. By adjusting sensory precision parameters, the model successfully simulates individualised patient recovery trajectories, exemplifying the controllability of lesion simulation and the core principle of mechanistic modelling (Sajid et al., 2020).

The third category addresses translation deficits. Models of this type attend to the specific difficulties patients exhibit during active, bidirectional translation tasks. These include an inability to accurately render words or sentences from one language into another as required, or the manifestation of asymmetrical translation capabilities between the two languages. Only a single study, S7, falls within this category (Sajid et al., 2020), and Figure 7 depicts the translation task performance of this model. At the level of core mechanism simulation, such models typically conceptualise translation as a specialised language task. The core mechanisms they simulate involve the strength of cross-language lexical associations and the specific control resources requisite for the translation process. Model architectures often incorporate direct or indirect pathways from source language lexical items to their target language equivalents. Computational lesion operations encompass the following four levels. Cross-language association lesion involves attenuating the connection strength between L1 and L2 lexical entries. Translation-specific control lesion entails applying operations analogous to those used for general language control deficits, but targeted specifically at the translation task node. Asymmetric lesion involves applying differential degrees of lesioning to the association or control pathways for L1 → L2 versus L2 → L1 translation. Regarding model output and validation, the model’s performance, error types, and reaction times on translation tasks are compared against patients’ scores on translation assessments.

Across the 12 computational simulation studies, model construction and calibration exhibit three shared features: parameters are typically initialised from individual patients’ language histories; researchers can precisely manipulate the locus, extent, and severity of lesions and simulate the rehabilitation process; and the validation imperative centres on mechanistic simulation rather than behavioural fitting alone. In terms of technical implementation, impairment is simulated predominantly through systematic parameter adjustment; a smaller number of studies employ individualised parameter configuration or theory-driven architectural constraints to reflect inter-patient variability. Contemporary LLMs have yet to assume a role in mechanistic simulation; only S29 utilised an LLM as an auxiliary tool for speech recognition (Bao et al., 2025).

The evaluation of model efficacy confronts considerable methodological challenges. The majority of the 12 simulation studies provide only qualitative validation. Over half of the studies including S1, S2, S3, S5, S7, S30, and S42 did not report quantitative performance metrics (Grasemann et al., 2010, 2011, 2021; Kiran et al., 2013a; Peñaloza et al., 2019; Sajid et al., 2020, 2021). Among the simulation studies that did provide quantitative metrics, S29 reported a reduction in Word Error Rate (WER) exceeding 30% (Bao et al., 2025), S31 reported a semantic decoding accuracy of 88.1% (Li et al., 2024), and S34 reported a WER of 12.4% (Rajendran & Chandrasekar, 2024). Beyond the core simulation sample, empirical and assessment-oriented studies additionally contributed quantitative performance data: S6 reported F1 scores (treated language improvement (TLI): 0.767; cross-language generalization (CLG): 0.790) (Marte et al., 2025), and S33 reported a classification accuracy of 83.4%. This heterogeneity reflects the absence of a standardised evaluation framework within the field.

**Figure 8:**
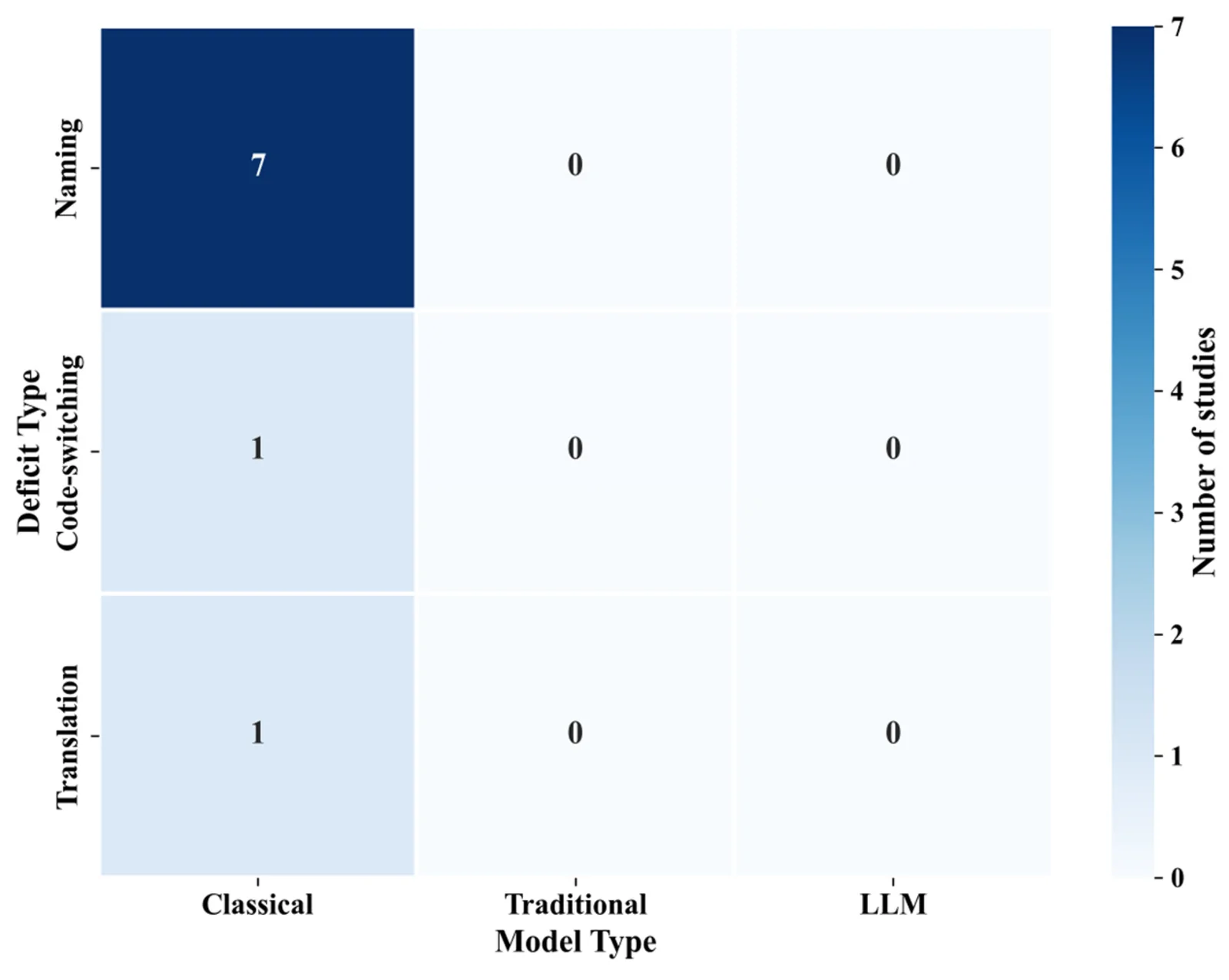
Comparison of simulation performance across deficit types. Note: S7 (Sajid et al., 2020) addresses both code-switching and translation deficits and is therefore represented in both categories. Column totals in this figure consequently exceed the number of unique studies.

Different simulation strategies serve distinct analytical purposes: parameter adjustment is suited to exploring generalised impairment effects, individualised configuration to inter-patient variability, and theory-architected models to direct testing of specific cognitive hypotheses. Traditional machine learning models oriented toward prediction demonstrate superior quantitative performance but typically do not elucidate underlying mechanisms, while LLMs have not yet been systematically harnessed for interpretable deficit simulation. A systematic cross-model comparison of convergent and divergent patterns is provided in Section 3.2.5. This current state of affairs is visually summarised in Figure 8.

#### 3.2.2. Computational Support in Clinical Assessment

The application of computational models in the assessment of bilingual aphasia is primarily manifested in two distinct roles: that of an automated analytical tool and that of a cognitive process quantifier. Drawing upon the 19 empirical studies and select computational simulation studies included in this review, as illustrated in Table 3, this section analyses the technical pathways, quantitative performance, and current limitations associated with these applications.

At the level of automated analysis, models are predominantly applied to pathological speech recognition and error type annotation. Fine-tuning pre-trained speech recognition models to accommodate pathological speech features can markedly enhance transcription accuracy for the speech of individuals with aphasia. Illustrative cases include a study within S29 that achieved a reduction in WER exceeding 30% (Bao et al., 2025), and the naming utterance verifier for aphasia (NUVA) tool in S33, which attained an automatic classification accuracy of 83.4% for pronunciation correctness in a naming task (Barbera et al., 2021). Core methodologies involve fine-tuning acoustic models on aphasic speech corpora and integrating language model constraints to bolster robustness against semantic paraphasias. Nevertheless, such approaches exhibit limited recognition rates for severe dysarthria or mixed errors and generally lack cross-linguistic recognition capabilities for bilingual code-switched speech.

With respect to automated error type annotation, technical implementations typically combine rule-based computation with machine learning classifiers to detect phonological or semantic paraphasias and to identify segments of language mixing. The study in S31 achieved a semantic category classification accuracy of 88.1% in an EEG semantic decoding task (Li et al., 2024). Limitations of such applications include low recall rates for low-frequency error types and, for the majority of studies, a lack of rigorous consistency comparison against the gold standard of annotations provided by speech-language pathologists (SLPs).

In its capacity as a cognitive process quantifier, the computational model can furnish latent variables that reflect internal processing dynamics, variables that are inaccessible through traditional assessment methods. By executing cognitive architecture models to simulate a patient’s naming process, researchers can quantify parameters such as the propagation delay of activation from semantic to lexical representations, the intensity of bilingual competition within the lexical layer, and the stability of language control nodes. Research documented in S30 has demonstrated that model-predicted lexical activation delays correlate significantly with patients’ actual naming latencies, and that metrics of competitive intensity computed by the model can aid in differentiating among aphasia subtypes (Peñaloza et al., 2019). In the context of modelling cross-language interference, researchers inject computational lesions into bilingual naming models to dynamically monitor the spillover activation levels of the non-target language and to compute language-specific inhibitory efficiency. These computational metrics have been empirically linked to patient clinical behaviour; for instance, activation spillover values exhibit a positive correlation with the frequency of spontaneous code-switching, while inhibitory efficiency metrics have been shown to predict the magnitude of improvement in language control following transcranial direct current stimulation (tDCS) interventions.

Despite this demonstrated potential, the current role of computational models in assessment is positioned as that of an adjunctive quantification tool rather than a standalone diagnostic system. All relevant studies necessitate integration with traditional clinical testing, with model outputs serving as supplementary quantitative indices. The limitations concerning the coverage of deficits are summarised in Table 3. Current applications confront several core methodological challenges. First, model training is heavily reliant on small-sample, high-quality annotated patient data, a constraint that restricts generalisability. Second, the overwhelming majority of evaluation studies are based on highly structured laboratory naming tasks, leaving the performance of models in analysing more ecologically valid naturalistic conversation largely unknown. Finally, existing tools exhibit a narrow linguistic coverage, being primarily tailored to mainstream language pairs such as English–Spanish, and lacking adaptability to a wider array of language pairings. These limitations collectively delineate the technical capabilities and application boundaries of computational models within the contemporary landscape of bilingual aphasia assessment.

#### 3.2.3. Treatment Prediction and Personalised Rehabilitation

The core breakthrough of computational models in the domain of bilingual aphasia treatment resides in their capacity to predict individualised recovery trajectories and optimise therapeutic parameters. Drawing upon four studies identified within the included literature, this research direction has coalesced into a discernible technical framework and has begun to accumulate clinical empirical evidence. The fundamental technical principle underlying predictive models lies in establishing a mapping function that links patient characteristics and treatment parameters to therapeutic outcomes, thereby enabling prospective prognostication. This is achieved through two complementary pathways. Simulation-based prediction via cognitive architectures operates by parameterising a patient’s initial neurocognitive state, simulating the repeated activation of target language lexical nodes during the therapeutic process, and ultimately generating predicted values for changes in naming accuracy. Such models adhere rigorously to principles of neuroplasticity, incorporating weight update rules to simulate the brain’s learning processes. Complementing this approach is the data-driven machine learning prediction pathway, exemplified by the work of Marte et al. (Marte et al., 2025). This method involves extracting multidimensional patient feature vectors and employing regression algorithms or classification models, such as Support Vector Machines (SVMs), to compute the probability of a patient achieving clinically significant improvement.

These models have demonstrated reliable predictive performance within prospective clinical cohorts. The BiLex model in S5, applied to retrospective naming therapy data from 13 bilingual individuals with aphasia, successfully simulated improvement in the treated language and captured individual variability in cross-language generalisation (Grasemann et al., 2021). The machine learning model in S6, trained on data from 48 patients, achieved F1 scores of 0.767 (TLI) and 0.790 (CLG) on the treatment response classification task, thereby substantiating its predictive capability within a heterogeneous population (Marte et al., 2025).

Furthermore, computational models are beginning to furnish decision support for treatment planning. S42 employed a generative model to simulate the mechanisms of functional recovery under varying lesion typologies, thereby providing a theoretical rationale for understanding the selection of therapeutic targets (Sajid et al., 2021). S14, through neuromodulation research, offered a preliminary exploration of the beneficial effects of cerebellar transcranial direct current stimulation (tDCS) on language and executive function (Coemans et al., 2025). The core value of current predictive optimisation models is thus juxtaposed with inherent potential risks, and these limitations collectively constitute the principal challenges that must be surmounted in transitioning from predictive validation toward generalised clinical application.

The preceding subsections have described the landscape of computational models across three functional domains. The following subsection applies the review’s three analytical instruments: the five-dimensional lesion coding scheme, the four-tier evidence hierarchy, and the seven-dimension methodological quality assessment, to the corpus.

#### 3.2.4. Application of the Analytical Frameworks

Applying the five-dimensional lesion coding scheme (Section 2.3.1, Table 2) to the 42 included publications revealed a marked concentration of lesion implementation at the parameter level. Among the 12 computational simulation studies, seven employed parameter-level lesions, predominantly through connection weight weakening, activation function alteration, or sensory precision reduction (S1, S2, S3, S5, S7, S27, S42). Control-level lesions were identified in only two studies: S7, which simulated weakened inhibitory control via reduced sensory precision within an active inference framework (Sajid et al., 2020), and S42, which implemented four distinct lesion types including control-level perturbations (Sajid et al., 2021). No study implemented an input/environmental-level lesion in isolation. Representational-level and architectural-level lesions were each observed in only one study (S42). Five of the 12 simulation studies (S29, S30, S31, S32, S34) employed no lesion method; S30 was initially double-coded as parameter-level and input/environmental-level but was revised to N/A across all five dimensions following consensus discussion, as it models healthy rather than lesioned bilingual processing (see Section 2.3). The remaining 30 publications (19 empirical studies and 11 review/methodology papers) were coded as not applicable, as they did not involve computational lesion implementation. A complete per-study coding is provided in Table S1 (Supplementary Materials).

Stratifying the 12 computational simulation studies according to the four-tier evidence hierarchy (Section 2.3.2) yielded a distribution concentrated at the lower tiers. No study attained Tier 3 (strong supportive evidence), which requires the satisfaction of three or more criteria among independent validation, differentiable prediction, quantitative goodness-of-fit, and specificity testing. Four studies reached Tier 2 (moderate indicative evidence): S5 (Grasemann et al., 2021), S7 (Sajid et al., 2020), S27 (Walker & Hickok, 2016), and S42 (Sajid et al., 2021). Six studies were classified as Tier 1 (weak suggestive evidence), which denotes weak suggestive evidence, typically meeting only the quantitative goodness-of-fit criterion, primarily through the reporting of accuracy, F1, or word error rate metrics, without accompanying independent validation or specificity testing. One study (S1) was classified as Tier 0 (descriptive fitting or hypothesis generation), providing qualitative description of model–behaviour congruence without meeting any of the four evidential criteria. Among the four Tier 2 studies, only S7 directly addresses bilingual-specific control mechanisms, namely, code-switching, translation, and language selection. The remaining three Tier 2 studies (S5, S27, S42) simulate naming or word repetition deficits, which, while clinically important, are not unique to bilingual populations. This distribution reflects a central finding of the present review: the computational modelling of bilingual-specific deficits currently rests on a single study. The complete per-study tier classification is available in Table S1. The remaining study (S30) was not assigned an evidence tier, as it does not implement a computational lesion.

As summarised in Table 4, the quality assessment revealed considerable variation across dimensions. Clinical/theoretical contextualisation (Q7) was a relative strength, with 26 of 42 studies rated High. By contrast, code and data accessibility (Q2) was predominantly rated Low (35 of 42 publications), and interpretation rigour (Q6) was rated Low for close to half of the included studies (17 of 42). These patterns identify specific areas where the field may direct efforts to strengthen methodological standards and computational reproducibility.

#### 3.2.5. Cross-Model Comparative Synthesis

Cross-model comparison of the 12 simulation studies reveals several convergent patterns that cut across technical pathways. First, parameter-level lesioning is the predominant implementation strategy: seven of the 12 simulation studies operationalise impairment through adjustment of connection weights, activation thresholds, or inhibitory strength, a pattern that holds across the BiLex family (Grasemann et al., 2011, 2021), SLAM (Walker & Hickok, 2016), and the active inference framework (Sajid et al., 2020, 2021). Second, competition and inhibition recur as core mechanistic constructs across theoretical frameworks, whether as lateral inhibition in connectionist architectures or as precision-weighted control in active inference models, suggesting that structurally analogous theoretical commitments about impaired bilingual control are being expressed in the formal vocabulary of different paradigms. Third, a shared limitation across paradigms is the predominance of behavioural fitting over mechanistic testing: even among studies attaining Tier 2 in the evidence hierarchy, success consists more reliably in reproducing known behavioural patterns than in subjecting specific cognitive hypotheses to exclusive test.

Beyond these convergences, existing models diverge along several dimensions. At the level of theoretical framework, BiLex and SLAM are rooted in the connectionist tradition and represent bilingual control as weight-based competition among lexical nodes; the active inference framework reconceptualises control as Bayesian balance between prior beliefs and sensory evidence, yielding accounts of the same clinical phenomena, such as translation asymmetries and alternating recovery, that differ in both mathematical structure and cognitive interpretation. The locus of bilingual control constitutes a second line of divergence: connectionist models locate language selection in lateral inhibitory connections at the lexical-semantic interface, whereas active inference places it at the level of higher-order, modality-independent control-state precision. Validation standards also diverge considerably: some models report quantitative goodness-of-fit indices, while the majority rely on qualitative description, with the result that comparable performance benchmarks across technical pathways are virtually absent. These divergences are not deficiencies of individual models but reflect a field whose theoretical foundations have yet to converge.

Distinguishing findings that are robust across paradigms from those that remain model-dependent yields the following picture. Robust findings include: (a) naming deficits can be simulated at the behavioural level through parameter-level attenuation of lexical-semantic mappings, without requiring the assumption of additional cognitive impairment; (b) cross-language generalisation, a phenomenon whereby treatment gains in one language transfer to the untreated language under specific conditions, has been independently reproduced in both the BiLex and active inference frameworks, albeit through different explanatory mechanisms (Grasemann et al., 2021; Sajid et al., 2020); and (c) the mapping between lesion operation and behavioural output is highly sensitive to prior parameter settings, a sensitivity that is itself a stable finding across paradigms. By contrast, model-dependent conclusions include the specific asymmetry patterns of cross-language recovery, the manner in which inhibitory imbalance manifests in code-switching deficits, and the directionality of translation asymmetries, all of which vary qualitatively with model architecture and parameterisation and cannot presently be regarded as robust knowledge accumulation in the field.

It is necessary to state plainly the current boundaries of cross-model comparative synthesis. The studies analysed in this review are not evenly distributed across a mature multi-model space; they are concentrated within the BiLex architecture and its variants. In the two deficit categories that define bilingual aphasia, namely code-switching and translation, the entire computational lesioning literature consists of a single independent study (S7) (Sajid et al., 2020), meaning that no second data point exists against which comparison could be drawn. This state of affairs is not a failure of the review method but one of its central empirical findings: computational modelling of bilingual-specific deficits currently operates at the level of single-point exploration and has not yet produced a multi-model competitive landscape within which systematic comparison can be conducted. It is this finding, rather than any particular inter-model comparison, that constitutes the review’s most important characterisation of the field’s current condition.

### 3.3. Integration of Research Themes and Status of Clinical Translation

An integrative analysis of the complete corpus of 42 publications reveals that the field of computational modelling in bilingual aphasia exhibits a tripartite structure. Specifically, empirical studies constitute the primary evidence base, computational simulation studies provide the impetus for mechanistic exploration, and review and methodological studies serve an integrative, framework-building function. Among these, empirical investigations grounded in patient behavioural data or clinical observation account for 19 publications (45.2%). Based on the distribution of study types, computational simulation studies comprise 12 publications (28.6%), while review articles and methodological studies aimed at theoretical synthesis and framework construction represent 11 publications (26.2%).

Computational simulation studies, which function as a critical nexus bridging theory and empirical observation, exhibit pronounced developmental asymmetry. In terms of the depth of mechanistic simulation, research targeting naming deficits has evolved into a relatively systematic modelling paradigm. Among the 12 computational simulation studies, a total of seven (58.3%) identify naming deficits as their core focus. Representative examples include the fine-grained parameterisation of lexical retrieval bottlenecks across studies in S2, S5, S30, employing the BiLex model (Grasemann et al., 2011, 2021; Peñaloza et al., 2019), and the mechanistic account of naming errors in conduction aphasia provided by the SLAM model in S27 (Walker & Hickok, 2016). In stark contrast, modelling endeavours directed toward deficits of greater bilingual specificity remain exceedingly sparse. Only a single study, S7, addressed the modelling of code-switching or language control (Sajid et al., 2020), an investigation that concurrently encompassed the simulation of translation deficits. This indicates that while the computational explication of naming impairments has reached a relatively mature stage, the mechanistic simulation of core bilingual phenomena, such as code-switching and translation, remains in a nascent state.

This imbalance in research focus is further mirrored by the fragmented and non-standardised nature of model performance evaluation. As documented in Section 3.2.1, over half of these studies provide only qualitative validation, and among those reporting quantitative metrics, no standardised evaluation framework has been established. Even among the few studies that do furnish quantitative metrics, the indices employed are highly heterogeneous. S6, as a machine learning application, reported F1 scores; S29 reported WER; S31 reported semantic decoding accuracy; S33, as an assessment tool, reported classification accuracy; and S34 reported WER. This heterogeneity in evaluation metrics renders direct comparison and robust validation across different models exceedingly difficult.

In the dimension of clinical translation, traditional behavioural intervention methods continue to predominate. The majority of the 19 empirical studies are descriptive or validation-oriented in nature. The application of computational models in clinical translation is concentrated within the domain of treatment response prediction. Representative work includes S5, wherein model predictions of individual recovery trajectories based on initial patient state parameters were found to correlate highly with actual improvement (Grasemann et al., 2021), and the prospective machine learning predictions in S6 (Marte et al., 2025). Additionally, computational simulation studies such as S1, S2, and S42 also address the simulation of therapeutic processes or the exploration of recovery mechanisms. However, based on the extant literature, no study to date has realised the dynamic, model-driven generation of a personalised rehabilitation protocol and completed its clinical validation. Temporal trend analysis indicates that computational simulation studies peaked at three publications in 2024, with new contributions also appearing in 2025; nevertheless, the overall volume remains lower than the growth observed for empirical studies and review articles. This pattern suggests that although mechanistic simulation research is steadily accumulating, a linear progression from model development to clinical implementation has yet to materialise.

Notably, despite rapid advancements in AI, classical computational models deeply engaged with specific cognitive mechanisms continue to dominate this research area. Among the 12 computational simulation studies, classical models account for eight publications (66.7%). Although preliminary forays involving LLMs in S29 and cutting-edge deep learning models in S31, S32, and S34 have been undertaken (Bao et al., 2025; Li et al., 2024; Ranjith & Chandrasekar, 2024; Rajendran & Chandrasekar, 2024), these efforts are predominantly concentrated on ancillary tasks such as speech recognition, signal decoding, or data augmentation, and have not yet been systematically applied to the core mechanistic modelling of bilingual control and impairment.

In summary, the field has established a relatively systematic computational-theoretical framework for the mechanistic explanation of naming deficits. However, the simulation of more complex deficits that inherently capture the essence of bilingualism, such as code-switching and translation, remains underdeveloped. A more fundamental issue is that existing models have yet to transcend the preliminary stage of predictive assistance, and a substantial chasm remains to be bridged before truly model-driven, closed-loop interventions can be realised. Addressing this chasm necessitates the concerted advancement of three parallel endeavours. First, a unified computational framework capable of encompassing a diverse array of bilingual deficits must be established. Second, a standardised system for evaluating model performance across different simulation tasks must be formulated to rectify the current deficiencies in quantitative validation and the prevailing confusion surrounding appropriate metrics. Third, priority should be accorded to the conduct of prospective, randomised controlled trials grounded in model-based predictions to rigorously ascertain their clinical utility.

The preceding section has reported the descriptive and analytical findings of the systematic review. The Discussion now interprets these findings, situating the evidence within the broader research landscape and examining its implications for theory, methodology, and clinical practice.

## 4. Discussion

### 4.1. Core Findings and Field Status

The systematic synthesis undertaken in this review elucidates the dual role assumed by computational models within bilingual aphasia research, as well as the specific developmental juncture at which the field currently resides.

On one hand, the core value of these models lies in furnishing a controlled experimental platform for theoretical exploration, a contribution most prominently manifested in the mechanistic simulation of language deficits. Through parametric manipulation, such investigations have successfully reproduced and predicted patient behavioural patterns, thereby circumventing certain limitations inherent in purely clinical observation and constituting the bedrock of contemporary theoretical advancement (Grasemann et al., 2011, 2021; Peñaloza et al., 2019, 2020). Nevertheless, most current work remains confined to the stage of behavioural fitting, where model outputs are aligned with clinical symptoms primarily through parameter adjustment, without advancing to mechanistic simulation. The latter entails directly testing cognitive neuroscience hypotheses by simulating lesions to specific neurocomputational circuits. This confinement consequently curtails the deep explanatory power of these models.

On the other hand, the research landscape is characterised by pronounced asymmetries. The integration of theoretical inquiry and clinical practice remains tenuous, and the translational potential of models to directly inform clinical assessment or therapeutic intervention is far from fully realised. Concurrently, research efforts have primarily focused on naming deficits, with less attention devoted to code-switching and translation deficits, which are more specific to the bilingual context (Kiran et al., 2013a; Kong et al., 2016; Sajid et al., 2020). In terms of technical methodology, the dominant paradigm continues to rely upon classical computational models and parameter adjustment, while a systematic evaluation framework is conspicuously absent. The majority of studies lack quantifiable performance metrics and depend primarily on qualitative description, thereby impeding cross-sectional comparison and robust validation across different models. Although emerging LLMs have begun to be explored, their application remains at a preliminary, ancillary stage (Bao et al., 2025), and they have yet to demonstrate systematic advantages at the core level of mechanistic simulation. It is worth noting a tension between the prominent role accorded to contemporary AI and large language model developments in the broader discourse surrounding computational aphasia research and the marginal representation of these approaches in the evidence base assembled by this review: only a single study among the 12 simulation studies employed an LLM, and that study used it as an auxiliary speech recognition tool rather than as a mechanistic simulation platform. This does not diminish the potential significance of LLMs for future work, but it indicates that their contribution to mechanistic understanding of bilingual aphasia remains, at the time of writing, a promissory note rather than an established research programme.

In summary, the field is presently confronting a critical transition from the replication of symptoms to the simulation of mechanisms. Advancing beyond this juncture necessitates not only a deepening of models’ capacity to simulate specific cognitive components but also a strengthening of their integration with clinical practice. Furthermore, the establishment of a more rigorous quantitative evaluation framework is imperative to fully realise the potential of computational models as instruments of translational research.

#### 4.1.1. Imbalanced Research Focus: Dominance of Naming Deficits vs. Underexplored Bilingual-Specific Deficits

The uneven distribution of research foci unveiled by this review is rooted in profound methodological and data-driven realities. The prominence of naming deficits as a focal point for mechanistic simulation is attributable, first, to the high degree of standardisation inherent in naming task paradigms, which facilitates their translation into model inputs and outputs. Second, such data are comparatively amenable to large-scale, systematic acquisition from clinical assessments, thereby furnishing a foundation for individualised model fitting. Third, mainstream connectionist models are architecturally predisposed to simulating the multi-level mappings among lexical, semantic, and phonological representations (Grasemann et al., 2011, 2021; Peñaloza et al., 2019), rendering the modelling of naming retrieval processes relatively tractable to implement. The field’s concentration on naming deficits also reflects its methodological inheritance from monolingual computational neuropsychology, where picture naming has long served as the canonical paradigm for lesion-simulation studies (Dell et al., 1997; Foygel & Dell, 2000; Plaut & Shallice, 1993; Welbourne & Lambon Ralph, 2005). In that tradition, the mapping between weight perturbations and naming error patterns is comparatively well characterised because the model architecture, a single-language lexical network, is relatively constrained. Extending this paradigm to bilingual-specific phenomena such as code-switching and translation requires modelling not only lexical access but also the cross-language control architecture that governs language selection, inhibition, and task switching, a more complex undertaking that, as the present review documents, has only begun to be attempted.

This convenience, however, has had an inescapable consequence: it has resulted in an insufficient grasp of precisely the bilingual nature that defines bilingual aphasia within extant research. Higher-order deficits, such as code-switching impairments, translation asymmetries, and syntactic mixing, reflect damage to mechanisms of cross-language cognitive control, monitoring, and task-schema switching, rather than isolated lexical retrieval failures. Such processes resist reduction to representational competition at the lexical level, yet existing research has, whether intentionally or inadvertently, simplified a complex, interactive bilingual control system into the superposition of damage to two relatively independent monolingual lexicons. While this simplification possesses operational value during the initial stages of investigation, it constrains a computational understanding of the genuine complexity inherent in bilingual aphasia (Sajid et al., 2020). Future research must shift the explanatory emphasis from relatively isolated lexical representation and retrieval processes toward the more dynamic cross-language control processes themselves, encompassing key mechanisms such as language selection, inhibition, switching, and monitoring.

#### 4.1.2. Language Pair Bias and Challenges to Theoretical Generalisability

Beyond the imbalance in research foci, another fundamental limitation identified by this review resides in the limited diversity of linguistic backgrounds. Nearly half of the 12 simulation studies are based on the English–Spanish language pair, a combination characterised by similar alphabetic writing systems and relatively simple morphological structures (Grasemann et al., 2010, 2011, 2021; Peñaloza et al., 2019, 2020). This bias engenders a gap in existing computational theories: a limited insight into how bilingual brains grappling with vastly different writing systems and morphological complexities respond to injury. A direct consequence is that the patterns of cross-language interference or recovery learned by current models may merely constitute idiosyncrasies specific to the properties of particular language pairs, thereby constraining the external validity and generalizability of these theories. Future computational modelling endeavours would benefit from incorporating linguistic typological variables as core parameters within both model construction and validation in order to develop a more generalisable computational account of bilingual cognitive control (Ranjith & Chandrasekar, 2024; Walker & Hickok, 2016).

The constraints imposed by this language-pair bias extend beyond a straightforward concern about external validity. English and Spanish share not only an alphabetic writing system but also membership in the Indo-European language family, a dominant subject-verb-object word order, structurally comparable article and pronoun systems, and code-switching norms that are sociolinguistically similar across the bilingual communities from which the primary data are drawn. This means that the “cross-language dynamics” learned by models calibrated predominantly on English–Spanish bilingual data, including the entire BiLex family, may capture regularities specific to this particular language pair rather than general properties of bilingual cognitive control. Transferring such models to pairings with greater typological distance, such as Mandarin–English, which pairs distinct writing systems with topic-prominent syntax, or Arabic–French, which crosses divergent writing directions and non-concatenative morphology, would require not merely re-estimating parameters but also rethinking which aspects of bilingual control are shaped by the proximity of particular language structures and which are cross-linguistically invariant. In this sense, the linguistic narrowness of the current evidence base is not a coverage gap that can be resolved by simply adding more language pairs to the sample; it raises the possibility that the field’s theoretical claims conflate patterns observed in one particular, typologically homogeneous set of bilinguals with general computational principles of bilingual aphasia.

The narrow linguistic coverage of the current evidence base raises a further question: to what extent are existing models applicable to other language types in principle? At the architectural level, the BiLex and active inference frameworks depend on core mechanisms such as competitive lexical activation, shared semantic representations, and inhibitory control circuits. These mechanisms are not intrinsically tied to properties of any specific language and are therefore capable, in principle, of being re-parameterised for different language pairs. At the practical level, however, such extension faces substantive challenges. For European languages such as Finnish–Swedish or German–Italian, inflectional morphological complexity and grammatical gender systems place demands on lexical representational granularity and control architecture that existing models have not been tested against. For Southeast Asian languages such as Thai, Vietnamese, or Tagalog, tonal systems, isolating morphology, and topic-prominent pragmatic structure introduce dimensions that have no counterpart in the English–Spanish modelling paradigm. Incorporating these languages into computational modelling would require not merely translating stimulus materials or substituting lexical entries but revisiting the architectural assumptions of bilingual control itself, specifically testing whether mechanisms assumed to be cross-linguistically invariant, such as inhibitory control and language selection, operate in the same manner in tonal or morphologically simplified languages. Any progress in these directions would constitute a stringent test of the generality claims of current computational theories.

#### 4.1.3. Heterogeneity of Bilingualism and Its Implications for Computational Modelling

The synthesis undertaken in this review exposes a deeper assumption running through the included literature: computational models have tended to treat “bilingual aphasia” as a relatively unitary diagnostic category. This simplification, while operationally necessary during early model development, stands in tension with what is known about the heterogeneity of bilingualism itself. Bilingualism is not a binary variable but a multidimensional continuum in which age of acquisition, language dominance, socio-linguistic environment, typological distance, literacy, code-switching norms, and degree of multilingualism each independently modulate how languages are organised in the brain and how they reorganise following injury (Luk & Bialystok, 2013). The following paragraphs examine the treatment, and more often the absence, of these variables in current computational models.

Age of acquisition. Age of acquisition is among the most important variables shaping how bilingual aphasia manifests and evolves. Kuzmina et al. found in a meta-analysis of 119 cases that bilinguals who acquired their second language before age seven showed comparable impairment across both languages following brain damage (Kuzmina et al., 2019), whereas later acquisition was associated with significantly worse performance in the less proficient language. Despite this evidence, existing computational models, whether within the BiLex family or the active inference framework, have not incorporated age of acquisition as a parameterisable variable, and their applicability to early bilinguals remains unexamined.

Language dominance and proficiency. Most computational models implicitly assume balanced bilingualism, yet in clinical reality pre-morbid language asymmetry is the norm: the majority of bilinguals have a dominant language, and the proficiency gap can be substantial. Following brain damage, dominance relationships may shift unpredictably, and cases in which the premorbidly dominant language is paradoxically more impaired are regularly documented. The application of a uniform lesion operation to a balanced versus a highly asymmetric system should, in principle, produce qualitatively different behavioural outcomes, yet this dimension has rarely been systematically explored. The models reviewed herein, calibrated predominantly on group-level data from balanced or near-balanced bilinguals, may not capture the impairment dynamics characteristic of highly asymmetric populations.

Sociolinguistic environment. Immersion settings, heritage-language contexts, and instructed second-language learning make different demands on the bilingual control system. Immersion contexts impose heavier daily requirements on language selection and inhibition and may shape different control strategies and patterns of vulnerability to brain damage (Green & Abutalebi, 2013; Grosjean, 1998). Existing computational models rely on data collected almost exclusively in North American and Western European clinical institutions, settings that typically correspond to immigrant or minority-language populations. Whether the mechanisms these models describe generalise to other sociolinguistic configurations, such as the multilingual ecology of Singapore, the diglossic Mandarin-dialect bilingualism of China, or the institutional multilingualism of Switzerland, remains an open question.

Typological distance. Whereas Section 4.1.2 discussed the language-pair bias from the perspective of theoretical generalisability, the present section addresses it as a dimension of bilingual heterogeneity. The English–Spanish pairing that dominates existing simulation studies shares an alphabetic writing system and relatively simple inflectional morphology, yet the typological spectrum of bilingualism extends considerably further. Parameter settings calibrated on English–Spanish data, such as the relative weighting of shared semantic representations, may not transfer to pairings with greater typological distance. What current models have captured may prove to be specific to particular language-pair combinations rather than general properties of bilingual cognitive control.

Literacy, code-switching norms, and multilingual variability. Beyond the variables discussed above, at least three further dimensions warrant attention. First, pre-morbid literacy, particularly among low-literacy or non-literate bilinguals, is associated with patterns of language organisation that differ from text-mediated, standardised models, and this population represents a substantial proportion of clinical aphasia caseloads. Second, code-switching practices vary substantially across communities. Dense code-switching environments, exemplified by the Puerto Rican Spanish–English community studied by Poplack (1980), and language-separation environments embody different psycholinguistic realities that may be amplified under pathological conditions. Third, a considerable proportion of individuals with aphasia use three or more languages, yet every computational simulation study in this review is restricted to the bilingual case. The extension to trilingual and multilingual systems introduces additional control dimensions, such as hierarchical language selection and graded inhibition, that place qualitatively new demands on model architecture (Goral & Hejazi, 2021). Taken together, the heterogeneity of bilingualism is not a peripheral background variable but a central methodological challenge for computational modelling. When a model is calibrated on one specific bilingual subpopulation, the scope of its claims must be bounded. This is not to diminish the value of existing work: detailed modelling of particular language pairs and deficit types under tightly controlled conditions is a necessary step. However, future research must elevate key dimensions of bilingual heterogeneity, including at minimum age of acquisition, language dominance, typological distance, and sociolinguistic environment, from implicit background noise to model parameters, and must test their influence through cross-population validation. Until this transition is accomplished, what current computational models depict is not the full landscape of bilingual aphasia but a set of localised delineations drawn from highly specific subpopulations.

### 4.2. Positioning This Review in Relation to Existing Literature

The positioning of this review vis-à-vis the extant literature reveals a multi-layered set of relationships. In contrast to studies oriented toward charting the prospective applications or technical inventories of AI, this review shifts the focus upstream toward the theoretical role of computational models as controlled, in-silico experimental platforms. Rather than cataloguing tools, it adopts the perspective of computational isomorphism to trace how modelling studies aimed at simulating bilingual aphasia mechanisms construct, manipulate, and validate their computational lesions. With respect to foundational classical modelling, the suite of studies exemplified by the BiLex model carries significant theoretical weight within the field. Through parameterisation of activation and inhibition relationships within the bilingual lexicon, S2, S5, and S30 have systematically explored patterns of naming deficits and cross-language generalization (Grasemann et al., 2011, 2021; Peñaloza et al., 2019). The existing literature frequently cites these investigations as illustrative successes, yet this review presses the inquiry further: to what extent do these simulations engage with the computational representation of language control mechanisms, or do they primarily accomplish behavioural fitting? A comparison between BiLex and the monolingual SLAM model in S27 (Walker & Hickok, 2016) reveals that some studies have endeavoured to encode multi-level phonological-lexical-semantic mappings within model architectures. Yet in the domain of cross-language control, most simulations continue to collapse inhibitory and selective processes into a small number of abstract parameters, leaving a considerable distance from the goal of progressing from behavioural fitting toward verifiable encoding of cognitive components.

A second notable category comprises modelling studies that pursue mechanistic explication with greater explicitness. Diverging from BiLex’s focus on behavioural fitting of naming deficits, S7 employed an active inference framework to simulate paradoxical translation and alternating recovery patterns (Sajid et al., 2020), while S42 utilised a generative model to dissect the synergistic competition between default and backup systems during word repetition tasks (Sajid et al., 2021). The significance of these contributions resides not in superior predictive accuracy but in their capacity to reify the failure of specific cognitive circuits as computable interventions through manipulation of parameters such as sensory precision and experience-dependent plasticity. This constitutes an instantiation of the standard of cognitive isomorphism advocated by this review: that the lesioned processes internal to the model correspond, in a one-to-one manner, with constructs within neurocognitive theory.

In relation to empirical case literature, investigations such as S11, S12, S20, and S21 have meticulously documented complex phenomena including asymmetric cross-language recovery in trilinguals, pathological code-switching, language mixing, and pragmatic dysregulation (Balasubramanian et al., 2026; Hameau et al., 2023; Miller Amberber, 2012; Peñaloza et al., 2025). Although these studies do not themselves incorporate computational models, they furnish detailed target phenomena for subsequent simulation efforts. S23 and S26 (Goral et al., 2019; Lerman et al., 2025) quantitatively analysed the frequency and embedding patterns of language mixing in multilingual aphasic narratives, corroborating predictions from certain language control theories without systematic computational modelling. This review posits that future work cannot be confined to fitting naming accuracy or paraphasia rates; higher-order cross-language behavioural patterns must become the subjects of in-silico experiments, fostering a tighter relationship between mechanistic simulation and naturalistic corpus analysis.

With respect to assessment and clinical application, this review establishes constructive points of convergence with tool-oriented development studies. Work such as S29, S33, and S34 has demonstrated the potential of traditional machine learning and deep learning in pathological speech recognition and automated annotation of naming errors (Bao et al., 2025; Barbera et al., 2021; Rajendran & Chandrasekar, 2024). These models currently surpass most classical cognitive models in technical performance, yet they lack an explicit representational framework for bilingual control and impairment mechanisms. Conversely, mechanism-oriented models offer greater explanatory depth but lag in automated processing of naturalistic speech. This review therefore advances a synergistic proposition: data-driven models could serve as front-end processors for feature extraction from high-dimensional inputs, while mechanism models conduct causal hypothesis testing within lower-dimensional, structured state spaces. Although this proposition remains to be systematically articulated in the literature, it constitutes a direct response to the central concern of this review: how computational models can genuinely inform clinical decision-making. Furthermore, this review aligns with literature concerned with methodological and theoretical frameworks. S25 elucidated the potential computational architecture of bilingual aphasia from the perspectives of population coding and parallel distributed processing (Nadeau, 2019), furnishing an abstract framework for interpreting multilingual impairment patterns without an operationalisable model tailored to specific tasks. A series of reviews and scoping reviews (S35–S39) have summarised the opportunities and ethical considerations surrounding AI in aphasia assessment and rehabilitation at a higher level of abstraction, yet they rarely engage with the concrete implementation pathways of computational lesions. In contrast, this review extends the same concerns downward by one level of analysis: it directly scrutinises the actual performance of specific modelling studies in terms of transparency, reproducibility, quantitative validation, and clinical relevance, thereby translating abstract principles into discernible variations in practice. In summation, the unique contribution of this review can be encapsulated across three dimensions. At the theoretical level, by introducing the perspectives of cognitive isomorphism and computational lesion experimentation, it re-anchors the conceptual role of computational models in bilingual aphasia research. At the evidentiary level, through systematic articulation of mechanistic models, machine learning tools, and a corpus of case and small-sample empirical studies, it illuminates both convergences and divergences between current modelling efforts and clinical phenomena. At the methodological level, through appraisal of transparency, data foundations, and quantitative validation, it elevates scattered reflections within the field into a collective agenda. In this sense, this review does not merely restate extant work but constructs a common frame of reference, one capacious enough to accommodate BiLex, active inference frameworks, SLAM, and deep learning tools alike, intended to empower subsequent investigations to more consciously navigate the competing demands of explanatory power, predictive capacity, and clinical utility.

### 4.3. Strengths, Consensus, and Limitations of Current Research

Computational models in bilingual aphasia research currently exhibit both unique potential and confront fundamental challenges. Their principal advantage resides in furnishing a highly controllable, in-silico experimental platform for understanding bilingual aphasia, thereby transcending the static constraints inherent in traditional clinical observation. By lesioning specific cognitive pathways within a model, researchers can systematically test theoretical hypotheses about core mechanisms, such as bilingual control and lexical retrieval, in a reproducible manner that goes beyond the ethical and practical boundaries of traditional clinical experimentation. For example, through parametric manipulation, classical models can not only fit the behavioural manifestations of naming deficits across varying degrees of severity but also predict treatment response patterns, including cross-language generalisation (Abutalebi & Green, 2007; Galletta et al., 2015). This capacity has fostered a fundamental consensus within the field: computational models constructed upon specific cognitive theories have demonstrated preliminary value in both explaining and predicting behavioural data. The boundaries of this consensus, however, also serve to delineate with clarity the core limitations of current research.

First, the validation foundation and explanatory depth of models remain inadequate. The majority of studies are still confined to the stage of behavioural fitting, wherein their success is more aptly characterised as the replication of symptoms rather than the achievement of mechanistic simulation. The correspondence between the computational lesions implemented and cognitive theories, such as inhibitory control, tends to be descriptive rather than encoded through a verifiable model architecture. This attenuates the potential of such models to propel theoretical advancement, rendering them, at times, more akin to a data-fitting “black box”. Concurrently, a scarcity of high-quality, longitudinal clinical data persists (Kohnert, 2004; Mooijman et al., 2022; Russell-Meill et al., 2025), with numerous models reliant upon theoretical derivation or calibration against small-sample data, thereby severely constraining both ecological validity and the precision of individualised prediction. This limitation is compounded by the nature of the behavioural data on which current models are trained and validated: the great majority of studies rely on constrained laboratory tasks, such as confrontation naming, word repetition, and controlled translation, that capture only a narrow slice of the communicative repertoire of bilingual speakers with aphasia. Spontaneous bilingual interaction, naturalistic code-switching, and discourse-level language control, which arguably represent the ecologically central manifestations of bilingual aphasia, remain absent from the modelling literature. Bridging this gap will require not only richer corpora but also modelling architectures capable of operating over the unstructured, interactive, and contextually embedded language use that characterises everyday bilingual communication.

Second, there exists a conspicuous narrowness in research focus and linguistic coverage. Investigative efforts have primarily focused on naming deficits, with less exploration devoted to code-switching and translation deficits, which are more representative of the bilingual context. (Calabria et al., 2019; Green & Abutalebi, 2013). Simultaneously, models exhibit a pronounced dependency on a limited set of language pairs, a factor that restricts the theoretical generalizability of research findings. Clinical translation of these models remains at an early stage; the aforementioned limitations have collectively precluded the maturation of any computational model into a routine clinical assessment or intervention tool, and the translational pathway from in-silico experimental insights to bedside application remains largely uncharted.

Third, the correspondence between the computational mechanisms proposed by current models and actual neurobiological architecture remains tenuous. The majority of models reviewed herein, including the connectionist architectures of the BiLex family, operate at the level of cognitive function, such as lexical selection, inhibitory control, and semantic mapping, and do not claim direct structural correspondence between model components and specific neural substrates. While this cognitive-level abstraction is both legitimate and productive for theoretical modelling, it entails that the “mechanisms” tested by these models should be understood as computational rather than neurobiological mechanisms. Translating a computational lesion finding, for instance, that a parametric reduction in inhibitory strength simulates a code-switching deficit, into a claim about dysfunction in a specific neural circuit has not been empirically established. This gap is not a deficiency of individual models but reflects the broader under-integration of computational modelling and systems neuroscience in the bilingual aphasia domain.

The model-agnostic stance adopted by this review also faces inherent tensions within its evaluative framework. Classical cognitive models, which prioritise theoretical interpretability, are more readily positioned to demonstrate potential in mechanistic simulation. Conversely, deep learning models, which pursue high predictive accuracy, are frequently disadvantaged in evaluations of mechanistic explanation owing to their “black-box” nature. This very tension underscores the reality that distinct modelling paradigms serve divergent research objectives. A key to future progress may reside in the construction of synergistic architectures. For instance, deep learning models could function as a front-end processor, handling high-dimensional naturalistic corpora and extracting structured behavioural features, which are subsequently fed into an interpretable back-end cognitive model for mechanistic simulation, thereby bridging the divide between predictive power and explanatory capacity (Zhang et al., 2025).

A related conceptual distinction that the field would benefit from maintaining more clearly is that among three types of model output. Behavioural simulation demonstrates that a model can reproduce an observed deficit pattern under specified conditions; it establishes plausibility but not necessity. Clinical outcome prediction forecasts individual patient trajectories or treatment responses, optimising for accuracy rather than theoretical insight. Explanation of cognitive architecture requires that the model’s internal components map onto independently motivated cognitive constructs in a manner that supports counterfactual testing. Much of the literature reviewed herein operates at the first level, gestures toward the third, and is occasionally evaluated against criteria appropriate to the second. Disentangling these three functions and matching evaluation criteria to the function a given model is designed to serve would clarify what individual studies can and cannot claim. Finally, it is imperative to subject this systematic review itself to critical scrutiny. Conducted in strict accordance with the PRISMA guidelines, this review undertook a comprehensive synthesis of computational modelling literature in bilingual aphasia specifically targeting mechanistic simulation. Grounded in a systematic analysis of the 42 included publications, this review inductively formulated operational instruments for the classification of computational lesions and the assessment of evidence strength, and provided a critical appraisal of the current state of the field. This work is intended to furnish methodological reference points for researchers, rather than to advance original theoretical contributions. Nevertheless, several non-negligible limitations must be acknowledged, and all findings are contingent upon the methodological quality of the included studies, a consideration that warrants careful attention when interpreting the conclusions.

First, literature coverage is subject to bias. To enhance feasibility and consistency, the search language for this review was restricted to English. This decision may have resulted in the omission of significant case studies or regional work published in other languages, such as Spanish or French. Language bias may influence the generalisability of the conclusions, thereby potentially underestimating the diversity of research perspectives to some extent. Second, the classification framework for computational lesions and the criteria for evidence strength assessment, while constructed to stimulate methodological discussion, require external validation in future research to ascertain their validity and generalizability. Third, owing to the high degree of heterogeneity among the included studies with respect to model types, lesioning methods, and evaluation metrics, a meta-analysis to provide a quantitative synthesis of effect sizes was not feasible. Consequently, the conclusions are principally grounded in qualitative synthesis and descriptive statistics. Furthermore, the reporting in a subset of primary studies was insufficiently detailed, posing challenges for data extraction and coding, and occasionally necessitating a degree of inference. Finally, to capture the most recent advances, conference papers and preprints were included; however, the evidentiary stability of such literature is comparatively lower, and conclusions drawn from them may be subject to revision upon subsequent formal publication.

Additionally, the inter-rater reliability analysis was conducted on a randomly selected subset of 10 publications (24% of the included studies) rather than on the full set of 42 studies. The resulting κ values (1.00 for lesion coding and evidence hierarchy; 0.65 for methodological quality assessment) indicate substantial to perfect agreement, but the precision of these estimates is inherently limited by the subsample size. In addition, the three databases were selected to jointly cover clinical, cognitive/linguistic, and computational modelling outlets relevant to this interdisciplinary topic: PubMed for clinical research, Web of Science for cognitive and linguistic research, and IEEE Xplore for computational modelling. We recognise that the exclusion of PsycINFO, Scopus, and Linguistics and Language Behavior Abstracts, or LLBA, constitutes a limitation. These databases index research on bilingualism and aphasia from behavioural, psycholinguistic, and linguistic perspectives, and their inclusion might have captured additional studies, particularly those employing behavioural methods adjacent to computational modelling. This limitation has been stated explicitly in Section 4.3, together with a note that the supplementary citation tracking and reference list searching described in Section 2.2 partially mitigated this restriction. Notwithstanding the aforementioned limitations, this review aspires that the knowledge map presented herein, the core challenges elucidated, and the future directions envisioned may provide a valuable point of reference for researchers across the fields of bilingual aphasia, computational cognitive science, and clinical speech-language pathology, thereby inspiring more rigorous, profound, and clinically attuned interdisciplinary exploration.

### 4.4. Key Unresolved Questions

A systematic synthesis of the extant evidence, coupled with a comprehensive appraisal of its methodological and theoretical contributions, reveals that while current computational modelling research in bilingual aphasia has yielded several instructive trajectories, a considerable distance remains from establishing a mature research paradigm that simultaneously possesses explanatory power, testability, and clinical translatability. Consequently, what this review brings to the fore is a constellation of interconnected core questions that await further elucidation through subsequent investigation.

First, the field is urgently confronted with the question of how the spectrum of models and technical pathways may be systematically compared. Current research encompasses a diverse array of paradigms, including classical cognitive neuropsychological models, connectionist architectures, generative models, active inference frameworks, traditional machine learning, and emerging LLMs. These models, however, exhibit marked divergence in terms of representational granularity, theoretical carrying capacity, methods of lesion implementation, and the tasks to which they are suited. Future research must therefore address the following two issues with greater precision: which types of bilingual aphasia phenomena are best simulated by different classes of models, respectively? And under what conditions do different computational lesioning techniques possess higher explanatory value, rather than merely superior fitting capacity?

Second, a question that remains insufficiently addressed within the field pertains to why bilingual-specific deficits have long occupied a peripheral position in existing simulations. Extant modelling efforts have focused on relatively general language deficits, such as naming impairments and lexical retrieval difficulties, while devoting less attention to code-switching deficits, translation impairments, imbalances in cross-language competition, and asymmetries in multilingual recovery. Ascertaining whether this imbalance originates from the difficulty of task construction, the paucity of data, the immaturity of theoretical frameworks, or structural limitations inherent in current models remains a priority for the field.

Several converging factors are likely to account for this asymmetry. First, the construction of ecologically valid experimental paradigms for code-switching and translation is methodologically demanding: code-switching is a spontaneous, context-sensitive behaviour whose laboratory elicitation often yields constrained and atypical samples, while translation tasks confound linguistic competence with executive demands in ways that are difficult to isolate. Second, the scarcity of large, systematically annotated bilingual corpora, particularly those capturing naturalistic code-switching in aphasic speech, deprives computational modellers of the quantitative training and validation data on which model development depends. Third, theoretical accounts of bilingual language control remain contested, with competing frameworks emphasising different loci and mechanisms of control; in the absence of theoretical consensus about what needs to be modelled, computational operationalisation becomes correspondingly diffuse. Fourth, longitudinal data tracing the evolution of code-switching and translation abilities from pre-morbid baselines through acute impairment to recovery are virtually non-existent, yet such data are essential for constraining the temporal dynamics of computational lesion models. These factors are mutually reinforcing: the absence of data discourages model development, and the absence of models reduces the incentive to collect the relevant data. Breaking this cycle will require coordinated investment in both corpus development and model construction, a point to which we return in Section 4.5.

Third, a more profound question concerns the extent to which the mechanistic explanations proffered by current research are tenable. Although numerous studies have successfully reproduced certain error patterns at the behavioural level, behavioural similarity does not automatically equate to mechanistic isomorphism. Future research must subject the following issues to more rigorous scrutiny: does a lesion operation within the model demonstrably correspond to the failure of a definable cognitive component? Do the results furnish evidence of independent validation, differentiable prediction, quantitative goodness-of-fit, and specificity-based exclusion? In the absence of such evidence, does so-called mechanistic simulation constitute merely a more elaborate form of phenomenological fitting, or can it substantively advance theoretical testing? Underlying these methodological questions is a conceptual issue that the present review can acknowledge but not resolve: the notions of mechanistic explanation, simulation, model validity, and abstraction that figure throughout this discussion have been the subject of sustained philosophical analysis in the literature on scientific modelling and explanation. The computational models reviewed herein operate at what would be characterised, in that literature, as a computational or functional level of description, and the relationship between this level and neurobiological implementation, as well as the criteria for warranting an explanation as “mechanistic”, remain matters of active philosophical debate. Acknowledging this conceptual context does not diminish the value of the modelling work reviewed but situates its claims within the appropriate epistemological framework.

Finally, the most pragmatically significant yet unresolved issue is how to bridge the translational chasm spanning from in-silico experimentation to clinical utility. At present, mechanistic models evince greater potential at the level of explanation, whereas data-driven models exhibit superiority at the level of performance; yet a stable and operationalizable synergistic architecture between the two has yet to coalesce. Future research must further delineate the requisite study designs, data foundations, validation standards, and interdisciplinary collaborative mechanisms that would enable computational models to generate usable, trustworthy, and equitable ancillary value within authentic clinical settings without sacrificing theoretical interpretability.

In this sense, the contribution of this review lies not merely in the consolidation of existing findings but, more critically, in crystallising the questions that the field must confront directly in its next phase of development (Zhang et al., 2025). It is precisely these unresolved questions that will determine whether future computational modelling research in bilingual aphasia remains confined to methodological demonstration and localised fitting, or whether it can mature into a robust research paradigm that bridges cognitive theory, pathological mechanism, and clinical decision-making.

### 4.5. Future Research Directions

Grounded in a critical appraisal of the contemporary research landscape, future work must transcend the preliminary validation of technical feasibility and endeavour to instigate a fundamental shift in the field’s research trajectory, elevating the computational model from a simulation tool predominantly engaged in phenomenological fitting to an integrated system capable of elucidating cognitive mechanisms and informing clinical decision-making. To advance toward this objective, this review proposes the following three interrelated strategic research directions.

#### 4.5.1. From Behavioural Fitting to Explainable Mechanistic Simulation

The core limitation of current research resides in the insufficient mechanistic explanation afforded by computational models. The key to future breakthroughs lies in the development of computational modelling capable of achieving explainable mechanistic simulation. This necessitates that researchers deeply encode specific cognitive theoretical frameworks into the intrinsic architecture or training objectives of models, rather than merely adjusting parameters to achieve behavioural fit. For instance, models should incorporate modular components that can be mapped onto language task schemas or language selection mechanisms. By precisely lesioning specific connections or dynamics between these components, researchers can simulate code-switching or translation deficits arising from failures in higher-order control circuits, thereby directly testing pertinent theoretical hypotheses. Concomitantly, interpretive tools for probing the internal states of models must be developed, rendering the model’s decision-making processes transparent and traceable to the investigator. This pathway aspires to achieve neuro-computational isomorphism, wherein the computational dynamics of the model bear a mappable correspondence to hypothesised neurobiological mechanisms, thereby elevating the model into a testable experimental platform for testing and refining cognitive theory.

#### 4.5.2. Predictive and Personalised Applications for Precision Rehabilitation

The ultimate clinical value of computational models resides in their predictive capacity. Future research should pivot its emphasis from retrospective simulation toward prospective prediction and personalised intervention. Specifically, investigations should prioritise the integration of multimodal data to construct machine learning models capable of forecasting individual patients’ response trajectories to diverse rehabilitation strategies (Peñaloza et al., 2020). Furthermore, the development of patient-specific digital twin models (Tao et al., 2022) could be advanced, enabling the pre-enactment and optimisation of personalised intervention protocols within a secure, controlled virtual environment, and facilitating the dynamic adaptation of training content. Such research must adhere to clinical translational research methodologies, validating clinical utility, operationalizability, and cost-effectiveness through rigorous feasibility studies and pilot randomised controlled trials (RCTs), rather than solely pursuing high predictive accuracy on historical datasets. Success in this direction would propel computational models toward genuine translation into practical tools supporting clinical decision-making.

#### 4.5.3. Cultivating a Research Ecosystem That Supports Interdisciplinary Integration and Open Science

The realisation of the aforementioned technical objectives hinges upon the establishment of a sustainable, interdisciplinary, and synergistic ecosystem. This necessitates the formation of institutionalised, deep collaboration among clinical neuroscientists, computational modelling experts, speech-language pathologists (SLPs), and patient representatives. The community should collaboratively strive to formulate standardised protocols for data acquisition and sharing specific to this domain, establish consensus guidelines for the reporting and evaluation of computational models, and prospectively address the ethical principles and equity frameworks pertinent to clinical application. Only through such deep, standardised collaboration can the field ensure that research designs are tightly anchored to authentic clinical questions, that algorithm development adheres to principles of transparency and reproducibility, and that the resulting outputs are ultimately positioned to garner trust and adoption within the clinical domain.

Through the systematic synthesis of the included literature, this review, from a model-inclusive perspective, has elucidated both the progress and the limitations characterising current research in terms of methodology and theoretical explanation. This integrated analysis furnishes an evidence base for comprehending the research landscape of the field (McClelland, 2010). Looking ahead, by concentrating efforts on explainable mechanistic simulation and predictive, personalised applications, and by advancing systematically with the support of a robust interdisciplinary ecosystem, computational modelling research holds the promise of realizing its full potential. This entails not only deepening the understanding of why bilingual aphasia occurs but, more importantly, providing evidence-based decision support for how more precise and efficacious rehabilitation interventions can be delivered.

## 5. Conclusions

Centred on how computational models simulate language deficits in bilingual aphasia, this review systematically synthesised 42 publications spanning computational simulation studies, empirical clinical investigations, and methodological reviews. The integrative analysis indicates that the field has consolidated a research pathway employing computational lesions, imposed upon model parameters, representations, or processing conditions, to probe failures in bilingual control, lexical retrieval, and cross-language recovery within a formalised experimental framework. This indicates that the value of computational models extends beyond auxiliary assessment or engineering applications to include their role as instruments of mechanistic simulation. Classical cognitive neuropsychological models and connectionist architectures constitute the dominant paradigms and the principal evidentiary foundation for understanding impairment mechanisms, having accumulated a body of work on naming deficits and cross-language generalisation. Traditional machine learning methods exhibit superior performance on engineering tasks such as automated assessment, yet their capacity for mechanistic explication of bilingual control remains limited. Emerging LLMs currently serve as ancillary tools and have yet to be integrated into core mechanistic modelling. More critically, the evidence assembled herein reveals that the field remains in transition from behavioural fitting toward mechanistic testing. Deficits that embody bilingual specificity, such as code-switching impairments, translation deficits, and failures of cross-language control, have yet to receive systematic modelling commensurate with their theoretical significance. The majority of studies can reproduce select clinical behavioural patterns, but deficiencies in independent validation, specificity testing, and quantitative goodness-of-fit limit the evidentiary strength of their mechanistic claims. Looking forward, this review posits that the crucial next step resides not in pursuing higher predictive performance or more complex algorithms, but in encoding the core constructs of bilingual cognitive control theory more clearly within model architectures and testing their explanatory power within a rigorous, transparent, and comparable validation framework. Progress will require narrowing the distance between theoretical simulation and clinical application through open data sharing, multicentre longitudinal studies, and synergistic integration of mechanistic and data-driven models. In summary, although the field continues to confront challenges, such as limited linguistic coverage, data scarcity, non-uniform evaluation standards, and protracted clinical translation, the evidence assembled herein demonstrates that computational models can serve as platforms for testing cognitive-mechanistic hypotheses and for bridging clinical phenomena with theoretical explanation. It is in this sense that computational modelling offers a research trajectory for understanding bilingual aphasia that merits sustained pursuit.

## Figures and Tables

**Table 1:** Search strings and results per database.

Database	Search Strategy	Result
**Primary Search**		
Web of Science	(“bilingual aphasia” OR “multilingual aphasia” OR (bilingual AND aphasia)) AND (“code-switching” OR “code mixing” OR translation OR “language deficit” OR anomia)	55
PubMed	(“bilingual aphasia” OR “multilingual aphasia” OR (bilingual AND aphasia)) AND (“code-switching” OR “code mixing” OR translation OR “language deficit” OR anomia)	75
**Supplementary Search**		
IEEE Xplore	(“bilingual aphasia” OR “multilingual aphasia” OR (bilingual AND aphasia)) AND (“large language model” OR LLM OR GPT OR BERT OR “transformer model” OR “computational model” OR “neural network” OR “deep learning”)	2
Litmaps	Citation network tracing: forward and backward search from seed articles identified via primary database results, using Litmaps automated literature mapping	14
**Total**		146

**Table 2:** A Five-Dimensional Coding Scheme for Implementing Computational Lesions.

Lesion Dimension	Operational Definition	Typical Technical Approaches	Simulated Cognitive/Neural Mechanisms
Parameter-level	Perturbation of a model’s internal parameters	Injection of weight noise; connection weakening; node inactivation; alteration of activation function thresholds	Simulates neuronal/synaptic dysfunction; diminished signal transmission efficiency due to neurotransmitter imbalance
Representational-level	Interference with or restriction of a model’s internal knowledge representations	Perturbation of semantic embedding spaces; corruption of cross-language mapping matrices; restriction of representational dimensionality	Simulates degradation of semantic memory; blurring of conceptual systems; disruption of cross-language lexical connections
Control-level	Intervention in language selection/switching/inhibition modules	Weakening of gating units; increasing task-switching costs; disruption of goal-language schema signals	Simulates damage to prefrontal-subcortical control networks, leading to difficulties in code-switching and failure of non-target language inhibition
Architectural-level	Direct alteration of model structure; removal or restriction of functional components	Removal of attention heads; restriction of working memory buffer capacity; deletion of specific network layers or pathways	Simulates focal brain region damage; constrained working memory capacity; disruption of information integration pathways
Input/Environmental-level	Manipulation of training or input data to simulate experiential or perceptual deficits	Omission of L2 input; corpus biasing; input noise or information masking	Simulates imbalanced language acquisition; language experience deprivation; primary perceptual damage to auditory/visual input channels

Note: A single publication may employ a combination of multiple lesioning approaches.

**Table 3:** Contributions and Potential Risks of Computational Models in Bilingual Aphasia Applications.

Core Contribution	Representative Evidence	Potential Risk
Identification of high-benefit patient subgroups	[S6]: ML models predicted treatment response (F1 ≈ 0.77–0.79), identifying patients likely to benefit from therapy (Marte et al., 2025).	Small sample sizes may lead to model overfitting, limiting generalizability to new populations.
Quantification of cross-linguistic therapeutic targets	[S5]: The BiLex model, fitted to data from 13 patients, successfully simulated individual cross-language generalisation patterns (Grasemann et al., 2021).	Model complexity necessitates large volumes of high-quality clinical data for calibration, posing significant challenges for clinical translation.
Guidance of neuromodulation targets	[S14]: Cerebellar tDCS significantly enhanced inhibitory control and language recovery in bilingual patients (*p* < 0.05) (Coemans et al., 2025).	Inter-individual variability in brain anatomy affects the distribution of the stimulation field, potentially reducing the precision and consistency of tDCS intervention outcomes.
Optimisation of therapeutic resource allocation	[S6]: Models identified key predictors, providing a basis for selecting the language of treatment and avoiding ineffective interventions (Marte et al., 2025).	Model predictions have yet to integrate patients’ personal preferences and sociopragmatic needs, which may impact intervention acceptability and ecological validity.

**Table 4:** Summary of methodological quality assessment across the seven dimensions.

Dimension	Label	High	Medium	Low	N/A
Transparency and reproducibility of model description	Q1	8	15	19	0
Accessibility of code and data	Q2	3	4	35	0
Theoretical/empirical justification for lesioning method	Q3	3	7	1	31
Ecological validity of data utilised	Q4	24	2	14	2
Appropriateness of evaluation methods	Q5	17	19	6	0
Rigour of result interpretation	Q6	11	14	17	0
Depth of clinical/theoretical contextualisation	Q7	26	9	7	0

Note: Each dimension was rated on a three-point scale (High/Medium/Low). Per-study coding for all seven dimensions is provided in Table S1.

## Data Availability

No new data were generated in this systematic review. All extracted data supporting the reported results are included in the article and its Supplementary Materials. Further inquiries can be directed to the corresponding author upon reasonable request.

## Supplementary Materials

The following supporting information can be downloaded at: https://www.mdpi.com/article/10.3390/bs16091486/s1, Table S1: Characteristics and per-study coding of the 42 included publications.

## References

[B1-behavsci-16-01486] Abutalebi J., Green D. (2007). Bilingual language production: The neurocognition of language representation and control. Journal of Neurolinguistics.

[B2-behavsci-16-01486] Adikari A., Hernandez N., Alahakoon D., Rose M. L., Pierce J. E. (2024). From concept to practice: A scoping review of the application of AI to aphasia diagnosis and management. Disability and Rehabilitation.

[B3-behavsci-16-01486] Ansaldo A. I., Saidi L. G. (2014). Aphasia therapy in the age of globalization: Cross-linguistic therapy effects in bilingual aphasia. Behavioural Neurology.

[B4-behavsci-16-01486] Bak T. H., Nissan J. J., Allerhand M. M., Deary I. J. (2014). Does bilingualism influence cognitive aging?. Annals of Neurology.

[B5-behavsci-16-01486] Balasubramanian A., Hofweber J. E., Bose A. (2026). Cross-linguistic asymmetries in language production and code-switching patterns in bilingual aphasia. Cognitive Neuropsychology.

[B6-behavsci-16-01486] Bao C., Huo C., Chen Q., Gao C. (2025). AS-ASR: A lightweight framework for aphasia-specific automatic speech recognition. 2025 IEEE biomedical circuits and systems conference (BioCAS).

[B7-behavsci-16-01486] Barbera D. S., Huckvale M., Fleming V., Upton E., Coley-Fisher H., Doogan C., Shaw I., Latham W., Leff A. P., Crinion J. (2021). NUVA: A naming utterance verifier for aphasia treatment. Computer Speech & Language.

[B8-behavsci-16-01486] Calabria M., Grunden N., Serra M., García-Sánchez C., Costa A. (2019). Semantic processing in bilingual aphasia: Evidence of language dependency. Frontiers in Human Neuroscience.

[B9-behavsci-16-01486] Coemans S., Struys E., Tsapkini K., De Aguiar V., Engelborghs S., Bier J.-C., Paquier P., Keulen S. (2025). Modulating language and executive functions in bilingual aphasia with cerebellar tDCS: A case series. Brain and Language.

[B10-behavsci-16-01486] Coemans S., Struys E., Tsapkini K., Paquier P., Vandenborre D., Keulen S. (2023). Case report: The effects of cerebellar tDCS in bilingual post-stroke aphasia. Frontiers in Human Neuroscience.

[B11-behavsci-16-01486] Cooke A., Smith D., Booth A. (2012). Beyond PICO. Qualitative Health Research.

[B12-behavsci-16-01486] Dell G., Schwartz M., Martin N., Saffran E., Gagnon D. (1997). Lexical access in aphasic and nonaphasic speakers. Psychological Review.

[B13-behavsci-16-01486] Dijkstra T., van Heuven W. J. B. (2002). The architecture of the bilingual word recognition system: From identification to decision. Bilingualism: Language and Cognition.

[B14-behavsci-16-01486] Dijkstra T., Wahl A., Buytenhuijs F., Van Halem N., Al-Jibouri Z., De Korte M., Rekké S. (2019). Multilink: A computational model for bilingual word recognition and word translation. Bilingualism: Language and Cognition.

[B15-behavsci-16-01486] Feigin V. L., Forouzanfar M. H., Krishnamurthi R., Mensah G. A., Connor M., Bennett D. A., Moran A. E., Sacco R. L., Anderson L., Truelsen T., O’Donnell M., Venketasubramanian N., Barker-Collo S., Lawes C. M. M., Wang W., Shinohara Y., Witt E., Ezzati M., Naghavi M., Murray C. (2014). Global and regional burden of stroke during 1990–2010: Findings from the Global Burden of Disease study 2010. The Lancet.

[B16-behavsci-16-01486] Foygel D., Dell G. S. (2000). Models of impaired lexical access in speech production. Journal of Memory and Language.

[B17-behavsci-16-01486] Galletta E. E., Cancelli A., Cottone C., Simonelli I., Tecchio F., Bikson M., Marangolo P. (2015). Use of computational modeling to inform tDCS electrode montages for the promotion of language recovery in post-stroke aphasia. Brain Stimulation.

[B18-behavsci-16-01486] Goral M., Hejazi Z. (2021). Aphasia in multilingual patients. Current Neurology and Neuroscience Reports.

[B19-behavsci-16-01486] Goral M., Norvik M., Jensen B. U. (2019). Variation in language mixing in multilingual aphasia. Clinical Linguistics & Phonetics.

[B20-behavsci-16-01486] Grasemann U., Kiran S., Sandberg C., Miikkulainen R. (2010). Simulating bilingual aphasia: A novel computational model. Procedia—Social and Behavioral Sciences.

[B21-behavsci-16-01486] Grasemann U., Peñaloza C., Dekhtyar M., Miikkulainen R., Kiran S. (2021). Predicting language treatment response in bilingual aphasia using neural network-based patient models. Scientific Reports.

[B22-behavsci-16-01486] Grasemann U., Sandberg C., Kiran S., Miikkulainen R., Laaksonen J., Honkela T. (2011). Impairment and rehabilitation in bilingual aphasia: A SOM-based model. Advances in self-organizing maps.

[B23-behavsci-16-01486] Green D. W., Abutalebi J. (2013). Language control in bilinguals: The adaptive control hypothesis. Journal of Cognitive Psychology.

[B24-behavsci-16-01486] Grosjean F. (1998). Studying bilinguals: Methodological and conceptual issues. Bilingualism: Language and Cognition.

[B25-behavsci-16-01486] Gusenbauer M., Haddaway N. R. (2020). Which academic search systems are suitable for systematic reviews or meta-analyses? Evaluating retrieval qualities of Google Scholar, PubMed, and 26 other resources. Research Synthesis Methods.

[B26-behavsci-16-01486] Hameau S., Dmowski U., Nickels L. (2023). Factors affecting cross-language activation and language mixing in bilingual aphasia: A case study. Aphasiology.

[B27-behavsci-16-01486] Kiran S., Grasemann U., Sandberg C., Miikkulainen R. (2013a). A computational account of bilingual aphasia rehabilitation. Bilingualism: Language and Cognition.

[B28-behavsci-16-01486] Kiran S., Sandberg C., Gray T., Ascenso E., Kester E. (2013b). Rehabilitation in bilingual aphasia: Evidence for within and between-language generalization. American Journal of Speech-Language Pathology.

[B29-behavsci-16-01486] Kohnert K. (2004). Cognitive and cognate-based treatments for bilingual aphasia: A case study. Brain and Language.

[B30-behavsci-16-01486] Kong A. P.-H., Abutalebi J., Lam K. S.-Y., Weekes B. (2014). Executive and language control in the multilingual brain. Behavioural Neurology.

[B31-behavsci-16-01486] Kong A. P.-H., Lam P. H.-P., Ho D. W.-L., Lau J. K., Humphreys G. W., Riddoch J., Weekes B. (2016). The Hong Kong version of the Oxford Cognitive Screen (HK-OCS): Validation study for Cantonese-speaking chronic stroke survivors. Aging, Neuropsychology, and Cognition.

[B32-behavsci-16-01486] Kuzmina E., Goral M., Norvik M., Weekes B. S. (2019). What influences language impairment in bilingual aphasia? A meta-analytic review. Frontiers in Psychology.

[B33-behavsci-16-01486] Lee S., Faroqi-Shah Y. (2024). A meta-analysis of anomia treatment in bilingual aphasia: Within- and cross-language generalization and predictors of the treatment outcomes. Journal of Speech, Language, and Hearing Research.

[B34-behavsci-16-01486] Lee Y.-Y. (2022). Bilingualism, dementia, and the neurological mechanisms in between: The need for a more critical look into dementia subtypes. Frontiers in Aging Neuroscience.

[B35-behavsci-16-01486] Lerman A., Bihovsky A., Meir N., Ben-Shachar M., Goral M. (2025). Language mixing in people with aphasia: A cross-linguistic analysis using the 4M model. Brain and Language.

[B36-behavsci-16-01486] Li C., Liu Y., Li J., Miao Y., Liu J., Song L. (2024). Decoding bilingual EEG signals with complex semantics using adaptive graph attention convolutional network. IEEE Transactions on Neural Systems and Rehabilitation Engineering.

[B37-behavsci-16-01486] Luk G., Bialystok E. (2013). Bilingualism is not a categorical variable: Interaction between language proficiency and usage. Journal of Cognitive Psychology.

[B38-behavsci-16-01486] Marte M. J., Carpenter E., Scimeca M., Russell-Meill M., Peñaloza C., Grasemann U., Miikkulainen R., Kiran S. (2025). Machine learning predictions of recovery in bilingual poststroke aphasia: Aligning insights with clinical evidence. Stroke.

[B39-behavsci-16-01486] McClelland J. L. (2010). Emergence in cognitive science. Topics in Cognitive Science.

[B40-behavsci-16-01486] Miller Amberber A. (2012). Language intervention in French–English bilingual aphasia: Evidence of limited therapy transfer. Journal of Neurolinguistics.

[B41-behavsci-16-01486] Moher D., Liberati A., Tetzlaff J., Altman D. G., for the PRISMA Group (2009). Preferred reporting items for systematic reviews and meta-analyses: The PRISMA statement. BMJ.

[B42-behavsci-16-01486] Mooijman S., Schoonen R., Goral M., Roelofs A., Ruiter M. B. (2026). Why do bilingual speakers with aphasia alternate between languages? A study into their experiences and mixing patterns. Aphasiology.

[B43-behavsci-16-01486] Mooijman S., Schoonen R., Roelofs A., Ruiter M. B. (2022). Executive control in bilingual aphasia: A systematic review. Bilingualism: Language and Cognition.

[B44-behavsci-16-01486] Nadeau S. E. (2019). Bilingual aphasia: Explanations in population encoding. Journal of Neurolinguistics.

[B45-behavsci-16-01486] Page M. J., McKenzie J. E., Bossuyt P. M., Boutron I., Hoffmann T. C., Mulrow C. D., Shamseer L., Tetzlaff J. M., Akl E. A., Brennan S. E., Chou R., Glanville J., Grimshaw J. M., Hróbjartsson A., Lalu M. M., Li T., Loder E. W., Mayo-Wilson E., McDonald S., Moher D. (2021). The PRISMA 2020 statement: An updated guideline for reporting systematic reviews. BMJ.

[B46-behavsci-16-01486] Palminteri S., Wyart V., Koechlin E. (2017). The importance of falsification in computational cognitive modeling. Trends in Cognitive Sciences.

[B47-behavsci-16-01486] Peñaloza C., Dekhtyar M., Scimeca M., Carpenter E., Mukadam N., Kiran S. (2020). Predicting treatment outcomes for bilinguals with aphasia using computational modeling: Study protocol for the PROCoM randomised controlled trial. BMJ Open.

[B48-behavsci-16-01486] Peñaloza C., Grasemann U., Dekhtyar M., Miikkulainen R., Kiran S. (2019). BiLex: A computational approach to the effects of age of acquisition and language exposure on bilingual lexical access. Brain and Language.

[B49-behavsci-16-01486] Peñaloza C., Marte M. J., Billot A., Kiran S. (2025). Cross-language interaction during sequential anomia treatment in three languages: Evidence from a trilingual person with aphasia. Cortex.

[B50-behavsci-16-01486] Pisano F., Manfredini A., Castellano A., Caltagirone C., Marangolo P. (2022). Does executive function training impact on communication? A randomized controlled tDCS study on post-stroke aphasia. Brain Sciences.

[B51-behavsci-16-01486] Plaut D. C., Shallice T. (1993). Deep dyslexia: A case study of connectionist neuropsychology. Cognitive Neuropsychology.

[B52-behavsci-16-01486] Poplack S. (1980). Sometimes I’ll start a sentence in Spanish y termino en español: Toward a typology of code-switching. Linguistics.

[B53-behavsci-16-01486] Rajendran R., Chandrasekar A. (2024). Conv-transformer-based Jaya Gazelle optimization for speech intelligibility with aphasia. Signal, Image and Video Processing.

[B54-behavsci-16-01486] Ranjith R., Chandrasekar A. (2024). GTSO: Gradient tangent search optimization enabled voice transformer with speech intelligibility for aphasia. Computer Speech & Language.

[B55-behavsci-16-01486] Russell-Meill M., Marte M. J., Carpenter E., Kiran S. (2025). Navigating the complexity of bilingual aphasia: Current insights and future directions. Brain Sciences.

[B56-behavsci-16-01486] Sajid N., Friston K. J., Ekert J. O., Price C. J., Green D. W. (2020). Neuromodulatory control and language recovery in bilingual aphasia: An active inference approach. Behavioral Sciences.

[B57-behavsci-16-01486] Sajid N., Holmes E., Hope T. M., Fountas Z., Price C. J., Friston K. J. (2021). Simulating lesion-dependent functional recovery mechanisms. Scientific Reports.

[B58-behavsci-16-01486] Senadheera I., Hettiarachchi P., Haslam B., Nawaratne R., Sheehan J., Lockwood K. J., Alahakoon D., Carey L. M. (2024). AI applications in adult stroke recovery and rehabilitation: A scoping review using AI. Sensors.

[B59-behavsci-16-01486] Tao F., Xiao B., Qi Q., Cheng J., Ji P. (2022). Digital twin modeling. Journal of Manufacturing Systems.

[B60-behavsci-16-01486] Tranfield D., Denyer D., Smart P. (2003). Towards a methodology for developing evidence-informed management knowledge by means of systematic review. British Journal of Management.

[B61-behavsci-16-01486] Tugwell P., Tovey D. (2021). PRISMA 2020. Journal of Clinical Epidemiology.

[B62-behavsci-16-01486] Walker G. M., Hickok G. (2016). Bridging computational approaches to speech production: The semantic–lexical–auditory–motor model (SLAM). Psychonomic Bulletin & Review.

[B63-behavsci-16-01486] Wang Y., Cheng W., Sufi F., Fang Q., Mahmoud S. S. (2024). A systematic review of using deep learning in aphasia: Challenges and future directions. Computers.

[B64-behavsci-16-01486] Welbourne S. R., Lambon Ralph M. A. (2005). Exploring the impact of plasticity-related recovery after brain damage in a connectionist model of single-word reading. Cognitive, Affective, & Behavioral Neuroscience.

[B65-behavsci-16-01486] Wohlin C. (2014). Guidelines for snowballing in systematic literature studies and a replication in software engineering. Proceedings of the 18th international conference on evaluation and assessment in software engineering.

[B66-behavsci-16-01486] Xu C., Liu Y., Wang X., Zhang C., Yao D., Yuan J. (2026). A review of large language model-driven speech disorder assessment methods. Computer Science.

[B67-behavsci-16-01486] Zhang Z., Ding X., Liang X., Zhou Y., Qin B., Liu T. (2025). Brain and cognitive science inspired deep learning: A comprehensive survey. IEEE Transactions on Knowledge and Data Engineering.

[B68-behavsci-16-01486] Zhong X. (2024). AI-assisted assessment and treatment of aphasia: A review. Frontiers in Public Health.

